# Precise base editing without unintended indels in human cells and mouse primary myoblasts

**DOI:** 10.1038/s12276-023-01128-4

**Published:** 2023-12-01

**Authors:** Da Eun Yoon, Na-Rae Kim, Soo-Ji Park, Tae Yeong Jeong, Bokkee Eun, Yongcheol Cho, Soo-Yeon Lim, Hyunji Lee, Je Kyoung Seong, Kyoungmi Kim

**Affiliations:** 1 Department of Physiology, Korea University College of Medicine, Seoul, 02841 Republic of Korea; 2 Department of Medicine, Korea University College of Medicine, Seoul, 02841 Republic of Korea; 3 Core Laboratory for Convergent Translational Research, Korea University College of Medicine, Seoul, 02841 Republic of Korea; 4 Department of Brain and Cognitive Sciences, Daegu Gyeongbuk Institute of Science and Technology (DGIST), Daegu, South Korea; 5 Korea Mouse Phenotyping Center, Seoul National University, 08826 Seoul, Republic of Korea; 6 Laboratory Animal Resource and Research Center, Korea Research Institute of Bioscience and Biotechnology, 28116 Cheongju, Republic of Korea; 7 Laboratory of Developmental Biology and Genomics, BK21 Program Plus for Advanced Veterinary Science, Research Institute for Veterinary Science, College of Veterinary Medicine, Seoul National University, 08826 Seoul, Republic of Korea; 8 Interdisciplinary Program for Bioinformatics, Program for Cancer Biology, BIO-MAX/N-Bio Institute, Seoul National University, 08826 Seoul, Republic of Korea

**Keywords:** Targeted gene repair, Genetics research, Gene expression

## Abstract

Base editors are powerful tools for making precise single-nucleotide changes in the genome. However, they can lead to unintended insertions and deletions at the target sites, which is a significant limitation for clinical applications. In this study, we aimed to eliminate unwanted indels at the target sites caused by various evolved base editors. Accordingly, we applied dead Cas9 instead of nickase Cas9 in the base editors to induce accurate substitutions without indels. Additionally, we tested the use of chromatin-modulating peptides in the base editors to improve nucleotide conversion efficiency. We found that using both dead Cas9 and chromatin-modulating peptides in base editing improved the nucleotide substitution efficiency without unintended indel mutations at the desired target sites in human cell lines and mouse primary myoblasts. Furthermore, the proposed scheme had fewer off-target effects than conventional base editors at the DNA level. These results indicate that the suggested approach is promising for the development of more accurate and safer base editing techniques for use in clinical applications.

## Revolutionising genome editing: new technique eliminates unwanted mutations

Researchers have found a method to enhance the precision of gene editing (manipulation of genes), lowering the probability of unwanted genetic changes. The research, directed by D.E.Y and K.K, reviewed the implementation of an enzyme system named CRISPR/Cas9, capable of being programmed to target certain genes in living organisms. Nevertheless, the researchers detected an issue: the enzyme occasionally led to unexpected insertions or removals in the DNA sequences at the target locations. To rectify this, the group substituted a component of the enzyme system with a ‘dead’ version that couldn’t alter DNA, and introduced chromatin-modulating peptide domains (protein parts that influence genetic material). This strategy eradicated unwanted genetic changes while preserving high editing effectiveness. This progress could contribute to safer and more exact gene editing in clinical and biological exploration. This summary was initially drafted using artificial intelligence, then revised and fact-checked by the author.

## Introduction

The clustered regularly interspaced short palindromic repeats (CRISPR)/Cas9 system is widely utilized in gene editing and continues to have expanded applications in various research areas. Bacterial adaptive immune-derived CRISPR/Cas9 has the ability to cleave DNA sequences at specific target sites guided by single guide RNAs (sgRNAs)^1^. Cas9/sgRNA-induced DNA double-strand breaks (DSBs) can be repaired by two major DNA repair mechanisms: nonhomologous end joining (NHEJ) and homology-directed repair (HDR)^2–7^. The NHEJ repair pathway causes insertions or deletions (indels) of DNA sequences at the Cas9/sgRNA-induced cleavage sites, resulting in simple knockout (KO) by frame shift. Although the HDR repair pathway offers precise DNA sequence replacement using specific donor DNA templates at the target sites, it is less efficient and can produce numerous indels simultaneously at the target sites. Additionally, HDR-mediated knockin (KI) generally occurs with high KI efficiency in the S/G2 phase of the cell cycle. This occurrence requires a homology arm (HA) of sufficient length on the donor DNA template, which limits the applicability of HDR-induced KI to nondividing cells^8–14^.

Base editing is an innovative gene editing method based on nickase Cas9 (nCas9) with the D10A variant and deaminase. These systems enable C-to-T nucleotide transitions by the cytosine base editor (CBE) or A-to-G nucleotide transitions by the adenine base editor (ABE) at the target sequence of the genome in an sgRNA-dependent manner^15,16^. Both base editors have specific activity windows on the target sequences that are located 13 to 17 nucleotides upstream of the protospacer adjacent motif (PAM)^15–18^. Base editing methods can accurately and efficiently rescue ~60% of known human pathogenic single-nucleotide polymorphisms (SNPs)^15,16,19,20^.

Various improved base editor versions have been built and reported by researchers to improve editing efficiency. AncBE4max and ABEmax are the representative evolved versions of the CBE and ABE, respectively, with improved nucleotide substitution efficiency via modified nuclear localization sequences (NLSs) and deaminase reconstructions^21^. Specifically, AncBE4max is reconstructed using bis-bpNLS and another ancestor deaminase, Anc689 APOBEC, with codon optimization, and ABEmax modifies the SV40 NLS to bis-bpNLS by introducing a codon-optimized deaminase.

An additionally developed dual-base editor using both adenine and cytosine deaminases can produce C-to-T and A-to-G conversions simultaneously^22–24^. The use of engineered Cas proteins applicable to various PAM sequences, such as Cas-NG and SpRY, can expand opportunities for target selection^25,26^. A new glycosylase base editor (CGBE) system containing uracil DNA N-glycosylase (UNG) capable of inducing C-to-G transversions at the desired target sites was reported subsequently^27,28^. This widens the scope of single-nucleotide substitutions, allowing the introduction of previously unacceptable mutations. In addition, a dual deaminase-mediated base editor (AGBE) in which ABE and CGBE are fused has also been reported^29^. This system can induce four types of nucleotide conversions (C-to-G, C-to-T, C-to-A, and A-to-G) simultaneously.

Previous studies have shown that base editors induce unwanted indels at the target sites in various cell types, such as mammalian cells and mouse embryos^15,30^. ABE8e, the most recently published and most effective ABE, also produces more of these unwanted indels^31,32^. For clinical application of the base editing system in gene therapy, it is crucial to specifically induce only desired nucleotide corrections at the target sequences without other mutations.

In this study, we applied various approaches to the base-editing system, such as using dead Cas9 (dCas9) instead of nCas9 and using chromatin-modulating peptide (CMP) domains, to achieve precise nucleotide substitutions at the desired target sites without indels. Such attempts may reduce the side effects of undesirable mutations arising from prior base editing, which is expected to contribute to more accurate and safer nucleotide-level editing in further clinical therapeutic studies.

## Materials and methods

### Cloning plasmid vectors for sgRNAs and base editors

Synthesized oligos were used for each of the target sgRNAs. The oligos were extended using Phusion polymerase (Thermo Fisher Scientific) and then ligated with the pRG2-GG vector (Addgene 104174) using T4 ligase (NEB). The cloned vector was transformed into competent DH5a cells (Invitrogen). The plasmids were extracted using a Midi Prep Kit (MACHEREY-NAGEL), and the sequences were confirmed by Sanger sequencing analysis (Bionics). Next, pCMV-BE3 (Addgene 73021), pCMV-AncBE4max (Addgene 112094), pCMV-ABE7.10 (Addgene 102919), and pCMV-ABEmax (Addgene 112095) were obtained from Addgene. The newly designed vectors containing dCas9 or CMPs or TadAmax of ABE8e were structured using the HiFi DNA Assembly Kit (NEB). The target sequences are listed in Supplementary Table 1.

### Cell culture and transfection

The cells were maintained in Dulbecco’s modified Eagle’s medium (DMEM; Welgene) supplemented with 10% fetal bovine serum (FBS; Gibco) for HEK293T (ATCC CRL-3216) and C2C12 (ATCC CRL-1772) or 10% bovine calf serum (BCS; Gibco) for NIH3T3 (ATCC CRL-1658) at 37 °C and 5% CO_2_. The cells were then seeded in 24-well plates (SPL) at 2 × 10^4^ cells per well. Approximately 16 h after plating, the cells were transfected with 750 ng of the base editor plasmid and 250 ng of sgRNA-containing plasmid with Lipofectamine 3000 (Thermo Fisher Scientific) according to the manufacturer’s protocols. After 72 h, the cells were collected, and the lysate or genomic DNA (gDNA) was used as the polymerase chain reaction (PCR) template for next-generation sequencing (NGS)^33^.

### Targeted deep sequencing

The target sites were amplified from gDNA using Phusion polymerase (Thermo Fisher Scientific) and the KAPA HiFi HotStart PCR Kit (Roche). The PCR amplicons were subjected to paired-end sequencing using the Illumina iSeq or MiSeq system (Illumina). Targeted deep sequencing data were then analyzed using the Cas-Analyzer program of the CRISPR RGEN tools (www.rgenome.net) or EUN program (eun-v2.com). All primers used are listed in Supplementary Table 2 and 3.

### Primary myoblast isolation

Fore-/hindlimb skeletal muscles were isolated individually from mice neonatal littermates. The primary myoblasts were grown at 37 °C with 5% CO_2_ in Ham’s F-10 medium (Welgene) supplemented with 10% cosmic calf serum (HyClone) and 5 ng/ml fibroblast growth factor (Peprotech)^34^. The sex and genotype of the *Dmd* KO or wild-type littermates were determined by PCR analyses of genomic DNA extracted from the tails of pups and analyzed by Sanger sequencing^35^.

### Primary myoblast culture and transfection

The isolated myoblasts were maintained in Ham’s F-10 medium (Welgene) with 10% cosmic calf serum, 50 ng/ml human basic fibroblast growth factor (bFGF; Peprotech), and 1% penicillin/streptomycin. We induced differentiation of the myoblasts to myotubes using 5% horse serum (Gibco) in DMEM and detected appropriate induction of differentiation through the mRNA expression levels of *Myh3* and *Dmd* by qPCR. The plates for the myoblast culture were coated with 0.01% sterile calf skin collagen (Sigma) in 0.2 N acetic acid (Sigma). Approximately 1 × 10^5^ myoblasts were plated on the collagen-coated 24-well plates with maintenance medium for 1 day. The next day, the medium was replaced with 500 µl of 5% horse serum in DMEM, and differentiation occurred for one day. Then, 2 h before transfection, the medium was replaced with 500 µl of DMEM with 2% horse serum for better transfection efficiency. Subsequently, 750 ng of the base editor vector and 250 ng of sgRNA-containing vector were transfected with JetPrime (PolyPlus) according to the manufacturer’s protocols. Finally, 24 h after transfection, the medium was replaced with 500 µl of 5% horse serum in DMEM, and fresh medium was replaced once every 48 h until harvest.

### RT‒qPCR

RNA was isolated from cultured myoblasts using the Mini BEST Universal RNA Extraction Kit (TaKaRa) and reverse transcribed to cDNA using the iScript™ cDNA Synthesis Kit (Bio-Rad) following the manufacturer’s instructions. qPCR was performed on a CFX96 system (Bio-Rad) using AccuPower® 2X GreenStar™ qPCR Master Mix (Bioneer) in triplicate. The expression level of each gene was determined with the ΔΔCq method and normalized to the mean Cq value of *Gapdh*. All qPCR primers used are listed in Supplementary Table 4.

### Off-target analysis

Off-target sequences were searched in the *Mus musculus* (mm10) genome using Cas OFFinder of CRISPR RGEN tools with mismatches for up to 3 base pairs^36^. The *Dmd* off-target candidates and primer sets used are listed in Supplementary Table 5–7.

### Statistics

Statistical analyses were performed using GraphPad Prism. The data are presented as the mean ± S.D. from at least 3–4 independent experiments, and significance was assessed using the unpaired Student’s *t*-test.

## Results

### Base editors cause unintended indels at the target sites

Several types of evolved base editors based on the CRISPR system have been developed for more accurate and efficient genome engineering^21,37^. Among these, AncBE4max and ABEmax were evolved by modifying codon usage, NLSs, and ancestral deaminase reconstructions^21^. These modifications greatly improve the efficiencies of these base editors in the cells, enabling effective SNP corrections^21^.

**Fig. 1:**
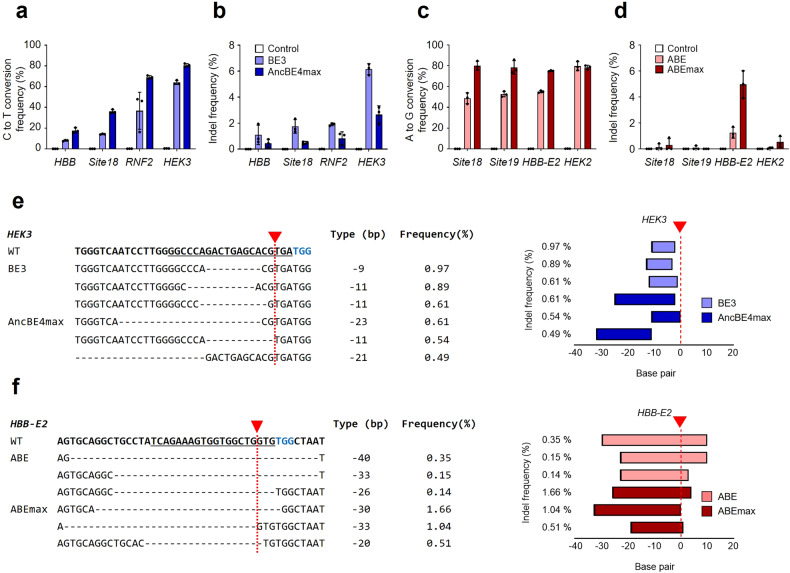
nCas9 in the base editor generated unintended indels at the target sites. **a**–**d** Comparisons of C-to-T or A-to-G substitutions and indel frequencies by BE3 and AncBE4max or ABE and ABEmax at each of four human target sequences in HEK293T cells. **e** Top three indel patterns in the *HEK3* target sequence induced by nCas9-based CBEs. **f** Top three indel patterns in the *HBB-E2* target sequence induced by nCas9-based ABEs. Red arrowheads and dotted lines, cleavage sites induced by SpCas9; blue letter, PAM sequence; underline, target sequences. Control indicates the untreated group. Each dot represents an individual target experiment. The data are shown as the mean ± S.D. of three independent experiments.

To determine whether various previously reported base editors (BE3, AncBE4max, ABE, and ABEmax) induce unwanted mutations in the target sequences, we selected four different human target genes and identified their base editing and indel efficiencies at the cellular level (Fig. 1a–f). As reported in previous studies, the nucleotide conversion efficiencies of enhanced AncBE4max and ABEmax were much higher than those of the other base editors (Fig. 1a, c). Among the CBE variants, AncBE4max exhibited the highest substitution efficiency of up to 82.2% at the *HEK3* target site while inducing lower indel efficiency than BE3 (Fig. 1a, b). In particular, indels were observed at frequencies up to 6.5% and 3.2% at the *HEK3* target sites for BE3 and AncBE4max, respectively, and most indels occurred at or near the cleavage site caused by SpCas9 (Fig. 1b, e). In the ABE system, ABEmax generally showed higher mutation levels of substitutions and indels than ABE (Fig. 1c, d). The ABE variants generally showed fewer indels relative to substitution efficiency than the CBE variants but still produced indels at the Cas9/sgRNA-induced cleavage sites (Fig. 1d, f). Our results demonstrate that the previously reported base editing systems induce multiple unintended indels at the DNA target sites.

### nCas9 results in indels at the target sites

**Fig. 2:**
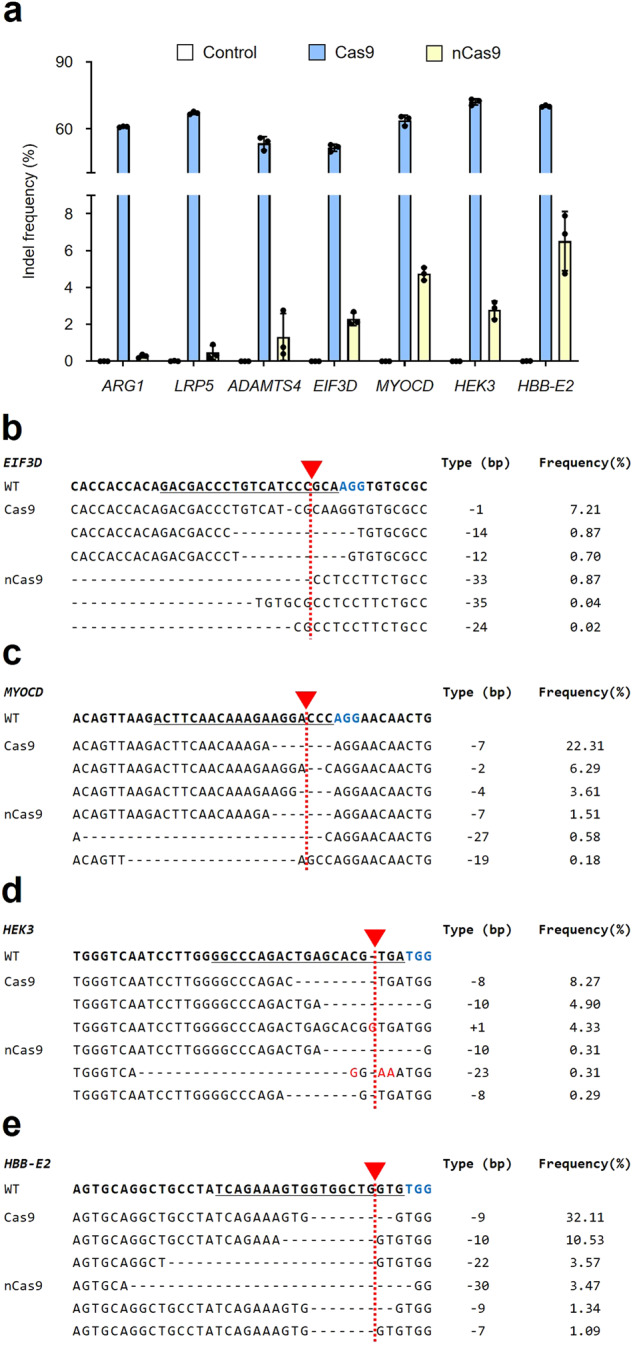
nCas9 induces indel mutations at the target sites. **a** Wild-type Cas9 and nCas9 generate indels at the seven target sites in human cells. **b**, **c**, **d**, **e** Alignments of the top three indel mutant sequences induced by Cas9 and nCas9 in representative *EIF3D*, *MYOCD*, *HEK3*, and *HBB-E2* targets. Red arrowheads and dotted lines, cleavage sites induced by SpCas9; red letter, insertion or substitution sequence; blue letter, PAM sequence; underline, target sequences. Control indicates the untreated group. Each dot represents an individual target experiment. The data are shown as the mean ± S.D. of three independent experiments.

We found that nCas9-based base editors induce certain numbers of indels at the target sites. According to some previous studies, DNA single-strand breaks (SSBs) can cause indel mutations via repair processes^38–40^. Therefore, we hypothesized that these unintended indels at the target sites caused by the base editors were generated by nCas9 (D10A). To determine whether the indels were indeed induced by nCas9, nCas9/sgRNA-targeting *ARG1*, *LRP5*, *ADAMTS4*, *EIF3D*, *MYOCD*, *HEK3*, or *HBB-E2* was delivered to human HEK293T cells, and the frequency of indels in the target sequences was analyzed. The results confirmed that up to 7.86% of indels occurred in the target sequences in the nCas9-treated group and that they had the same patterns as those induced by wild-type Cas9 (Fig. 2a–e). We found that nCas9 caused levels of indel mutations similar to those caused by the base editors at the desired target sites. This result suggests that most unintended indel mutations at the target sites were induced by nCas9 of the base editor.

### Unintended indels caused by base editors can be removed with the use of dCas9

**Fig. 3:**
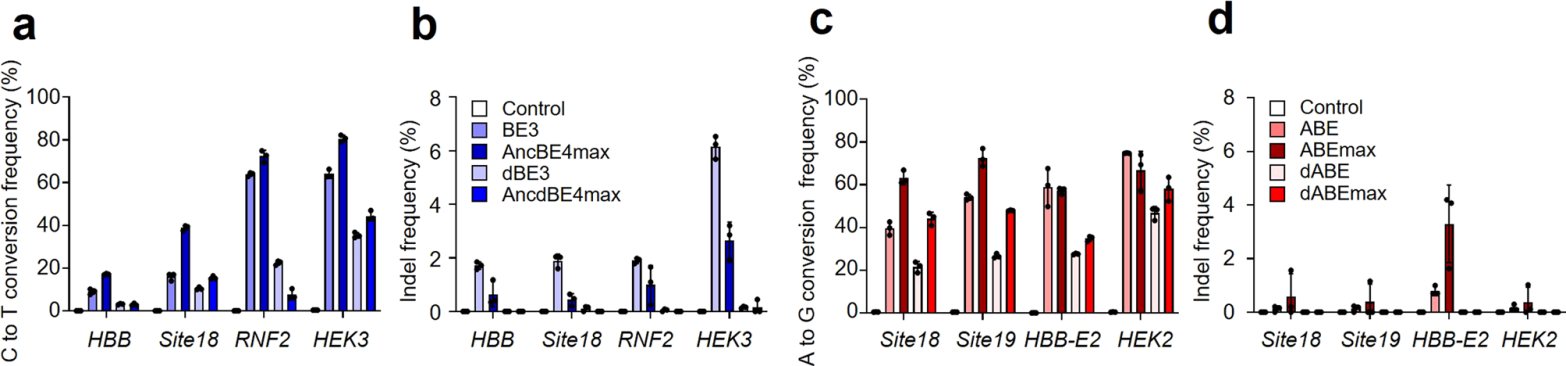
Unintended indels caused by base editors can be removed using dCas9. **a**, **b** Comparisons of C-to-T substitutions and indel frequencies by nCas9-based CBEs (BE3 and AncBE4max) and dCas9-based CBEs (dBE3 and AncdBE4max) at each of four human targets in HEK293T cells. **c**, **d** Comparisons of A-to-G substitutions and indel frequencies by nCas9-based ABEs (ABE and ABEmax) and dCas9-based ABEs (dABE and dABEmax) at each of four human targets in HEK293T cells. Control indicates the untreated group. The data are shown as the mean ± S.D. of four independent experiments. Each dot represents an individual target experiment.

To remove unwanted indels at the target site, nCas9 in the base editor was replaced with catalytically inactivated dCas9 (D10A and H840A) (Fig. 3a–d). Consequently, the unintended indels were mostly removed from all targets in both the CBE and ABE variants using dCas9 (dBE3, AncdBE4max, dABE, and dABEmax) (Fig. 3b, d). However, as described in a previous study^21^, the nucleotide conversion efficiency of C-to-T or A-to-G was also reduced simultaneously in all the targets (Fig. 3a, c). These data indicate that substitution of nCas9 with dCas9 could prevent unintended indels at the target sites; however, the reduction in the base editing efficiency caused by the use of dCas9 must also be addressed.

### Use of CMPs in dCas9-based base editing systems can improve substitution efficiency without causing indels

**Fig. 4:**
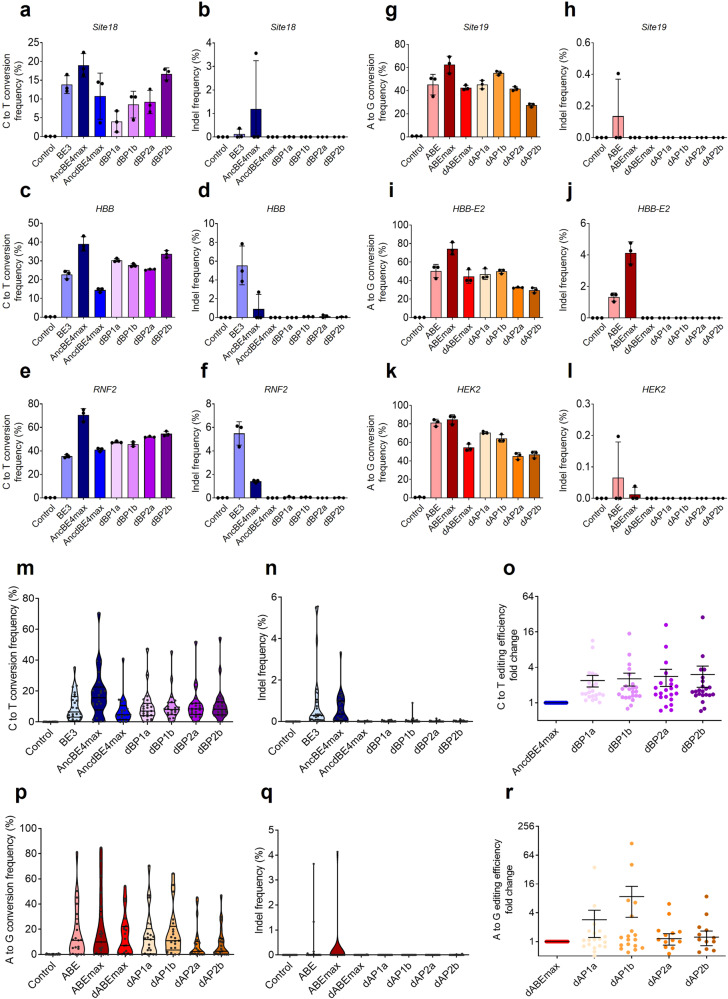
CMP-conjugated dCas9-based base editors promote nucleotide substitutions without unintended indels. **a**–**f** C-to-T substitutions and indel efficiencies of CBE and BP variants at Site18, HBB, and RNF2. **g**–**i** A-to-G conversions and indel frequencies of ABE and AP variants at Site19, HBB-E2, and HEK2. The data are shown as the mean ± S.D. of four independent experiments. Each dot represents an individual target experiment. **m**, **n** Base editing and indel frequencies of CMP-conjugated dCas9-based CBE variants at 23 target sites in HEK293T cells. **o** Fold changes in (**C**–**T**) conversions induced by all CMP-conjugated dCas9-based CBE variants at each target. **p**, **q** Comparisons of base editing and indel frequencies induced by CMP-conjugated dCas9-based ABE variants at 21 target sites in HEK293T cells. **r** Fold changes in (**A**–**G**) conversions of CMP-conjugated dCas9-based ABE variants at each target. Each dot represents an individual target experiment, and (**m**–**r**) include the means of the target values in (**a**–**l**). Control indicates the untreated group. The data are shown as the mean ± S.E.M. of the independent experiments in (**o**–**r**).

To improve the editing efficiency reduced by dCas9, we applied CMPs to the base editing system. CMPs can improve editing efficiency by unwinding the closed chromatin structures of the target sites^41^. Indeed, our previous study revealed that CMPs can open the closed chromatin structures of the target sites and improve the editing efficiencies in Prime editor^42^. We utilized human-derived high-mobility group nucleosome-binding domain 1 (HN1) and histone H1 central globular domain (H1G) as the CMP domains for dCas9-carrying base editors. To find the optimal locations of the CMP domains, HN1 and H1G were placed at various locations in the base editors, and their efficiencies were determined (Supplementary Fig. 1a). dBP2b was the most effective among the CMP-introduced CBE variants (dBP1a, dBP1b, dBP2a, and dBP2b), with a base editing efficiency comparable to or slightly lower than that of AncBE4max and complete elimination of unwanted indels (Fig. 4a–f).

Next, we generated CMP-introduced ABE variants (dAP1a, dAP1b, dAP2a, and dAP2b) using the same strategy as that for CBE using dCas9 and CMPs (Supplementary Fig. 1b). Similarly, none of the CMP-introduced ABE variants induced indels compared to ABE or ABEmax, and dAP1b showed the highest editing efficiency among CMP-introduced ABE variants (Fig. 4g–l). These CMP-introduced CBE or ABE variants were applied to 23 or 21 human target sites, respectively, to exclude targeting bias, and the ABE variants rarely induced indels compared to the CBE variants in most targets (Fig. m, n, p, q). The CMP-introduced CBE variants did not induce indels, except for one target in dBP1b (*HFE*; 0.9%). In contrast, BE3 or AncBE4max consistently generated indel mutations of up to 7.9% at the intended locations. Among the CMP-introduced CBE variants, dBP2b showed an average 3.0-fold increase in base editing efficiency compared with that of AncdBE4max; in particular, the efficiency improved by up to 28.4-fold at the *POU5F1* target (Fig. 4o). For dAP1b, the editing efficiency increased by an average of 8.8-fold compared with that of dABEmax, particularly with an up to 112.5-fold increase at the *ADAMTS4* target site (Fig. 4r). Although the CBE and ABE variants could not fully outperform their improved AncBE4max and ABEmax with regard to base editing efficiency, most of the unwanted indel mutations in the target sequences were eliminated. In most targets, neither ABE nor ABEmax induced as many indels as BE3 or AncBE4max (Fig. 4n, q). Additionally, the base editing efficiencies of dAP1a and dAP1b were lower than that of ABE or ABEmax but higher than that of dABEmax (Fig. 4q, r). As ABEmax exhibits high editing efficiency and low indel frequency, we tested whether introducing nCas9 instead of dCas9 into dAP1a (changes from dAP1a to nAP1a with the use of nCas9) and dAP1b (changes from dAP1b to nAP1b with the use of nCas9) would result in fewer indels and increased base editing efficiency compared to ABEmax (Supplementary Fig. 2a–c). Both nAP1a and nAP1b exhibited increased A-to-G substitution efficiencies owing to the use of nCas9 (Supplementary Fig. 2a). Specifically, the substitution efficiency of nAP1b was enhanced by up to 493.2-fold in 18 human gene targets compared with that of ABEmax (Supplementary Fig. 2c). However, indels also increased in three targets of nAP1a and four targets of nAP1b (Supplementary Fig. 2b). Collectively, our results suggest that using dCas9 and CMPs in base editing systems is necessary to achieve precise nucleotide conversions without undesirable indels.

ABE8e is one of the most recently evolved ABEs, and ABE8e, which maximizes editing efficiency, can induce many indels as well as high A-to-G substitution efficiency^31,32^. To eliminate unintended indels while maintaining high A-to-G substitution efficiency, we applied dCas9 and CMPs to ABE8e (dAP1b8e) (Supplementary Fig. 3). Our results showed that dAP1b8e produces an ~10–20% lower A-to-G conversion efficiency than ABE8e in five different human targets, but it is able to eliminate indel mutations (Supplementary Fig. 4a–j).

**Fig. 5:**
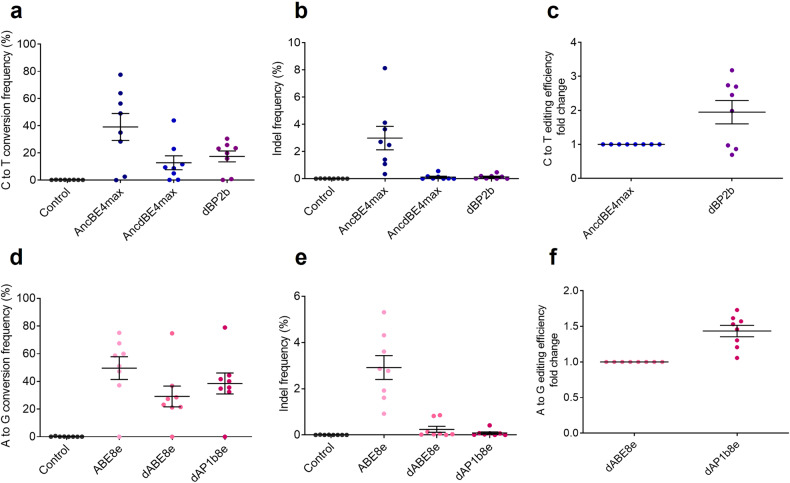
CMP-conjugated dCas9-based base editors enhance base editing efficiencies without undesired indels in mouse targets. **a**, **b** Comparisons of C-to-T conversions and indel efficiencies of AncBE4max, AncdBE4max, and dBP2b in eight mouse gene targets. **c** Fold change in the base conversion efficiency of dBP2b compared with AncdBE4max. **d**, **e** Efficiencies of A-to-G substitutions in CMP-conjugated or CMP-unconjugated ABE8e variants. **f** Fold change in base conversion efficiency of dAP1b8e compared with dABE8e. dAP1b8e promotes base editing efficiency at all targets in mouse Neuro2a. The data are shown as the mean ± S.E.M. of the independent experiments in (**c**, **f**). Each dot represents an individual target experiment. Control indicates the untreated group.

Since dBP2b and dAP1b8e have the highest nucleotide conversion efficiencies among the CMP base editor variants, we compared them with non-CMP conjugated base editors at eight targets in mouse cells. dBP2b increased the editing effect by an average of 2.0-fold (up to 3.1-fold) more than AncdBE4max, and dAP1b8e increased the effect by 1.4-fold (up to 1.7-fold) more than dABE8e without generating indels (Fig. 5). These data suggest that the dead base editor with CMPs generally works similarly in mice and humans.

### dBP2b and dAP1b8e efficiently induce base editing without unwanted indels at the target sequences in mouse primary myoblasts

**Fig. 6:**
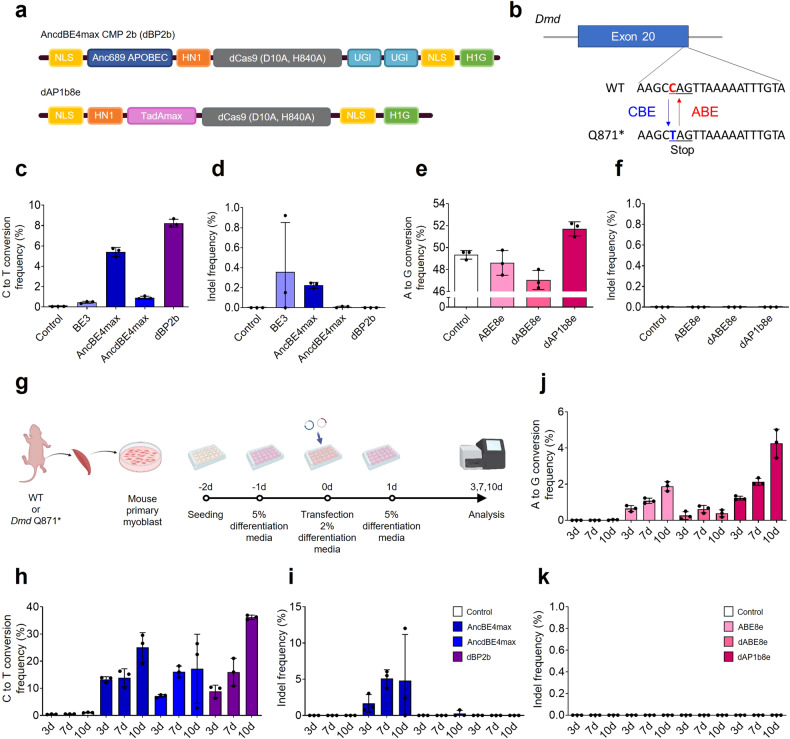
dBP2b and dAP1b8e base editors can induce high base editing efficiencies without indel mutations in primary myoblasts. **a** Construction of dBP2b and dAP1b8e. **b** Illustration of premature stop codon introduction by C-to-T conversion in exon 20 of the *Dmd* gene or rescue by A-to-G transition in exon 20 of *Dmd*. **c** Nucleotide substitution frequencies of CBE variants (BE3, AncBE4max, AncdBE4max, and dBP2b) in C2C12. **d** Indel frequencies of the BE3, AncBE4max, AncdBE4max, and dBP2b variants. **e** A-to-G conversion frequencies of ABE variants (ABE8e, dABE8e, and dAP1b8e) in *Dmd*^+/Q871*^ NIH3T3 cells. **f** Indels were not generated by any ABE variants. **g** Schematic outline of the mouse primary myoblast experiment. Primary myoblast cells were obtained from WT or *Dmd* Q871* neonatal skeletal muscle. The base editor and sgRNA-containing vectors were delivered to the cells on the second day of differentiation. **h**–**j** Identification of base substitutions and indel frequencies on days 3, 5, and 10 after transfection of CBE variants (AncBE4max, AncdBE4max, and dBP2b) and ABE variants (ABE82, dABE8e, and dAP1b82) in myoblast cells. **k** All ABE variants showed no indels. Control indicates the untreated group. The data are shown as the mean ± S.D. of three independent experiments. Each dot represents an individual target experiment.

To evaluate whether the base editing frequency could be improved without introducing unintended indel mutations, we tested the optimized dBP2b and dAP1b8e variants in mouse primary myoblast cells (Fig. 6a). We first designed a stop codon (CAG > TAG, Q871*) by C-to-T conversion at the end of mouse *Dystropin* (*Dmd*) exon 20 using CBE variants (BE3, AncBE4max, AncdBE4max, or dBP2b) and a *Dmd*-targeting sgRNA (Fig. 6b)^30^. By comparing the C-to-T conversion efficiencies of the CBE variants in the mouse myoblast line C2C12, it was found that dBP2b exhibited significantly higher editing efficiency than AncBE4max without causing indels (Fig. 6c, d). We also compared the A-to-G conversion and indel frequencies among ABE8e, dABE8e, and dAP1b8e by applying a strategy to restore the *Dmd* KO to normal in *Dmd*^+/Q871*^ NIH3T3 cells. The *Dmd*^+/Q871*^ NIH3T3 cell line has a C-to-T conversion at one allele in exon 20 of the mouse *Dmd* gene, resulting in a stop codon (Fig. 6b). We found that dAP1b8e induced A-to-G transitions more efficiently than ABE8e without any indel mutations in the *Dmd*^+/Q871*^ cells (Fig. 6e, f). Interestingly, both dBP2b and dAP1b8e showed higher base substitution efficiencies than the other CBE or ABE variants in mouse cells.

Then, as an alternative to in vivo experiments, we assessed the nucleotide substitution and indel efficiencies of the dBP2b and dAP1b8e variants using mouse primary myoblast cells. These cells were isolated from neonatal skeletal muscle of wild-type (WT) or *Dmd* Q871* KO mice within 5 days of birth (Supplementary Fig. 5). To verify that the desired base editing can be achieved accurately without unintended insertions or deletions even after long-term expression, we expressed the plasmids of dBP2b and dAP1b8e—the most optimized base editor variants in primary myoblasts—for up to 10 days (Fig. 6g; Supplementary Fig. 6). All CBE variants (AncBE4max, AncdBE4max, and dBP2b) gradually exhibited increasing C-to-T conversion efficiencies over time. Notably, dBP2b demonstrated the highest base editing efficiency, reaching up to 36.5% on day 10, without any unintended indels at the target sites (Fig. 6h, i). Similarly, dAP1b8e also showed a higher A-to-G substitution efficiency (4.3%) than ABE8e or dABE8e, and none of the ABE variants induced any indels (Fig. 6j, k). These results demonstrate that dBP2b and dAP1b8e are highly effective for achieving accurate and precise base editing without unintended indel mutations in primary myoblasts, even after long-term expression.

### dBP2b and dAP1b8e can induce low off-target effects

**Fig. 7:**
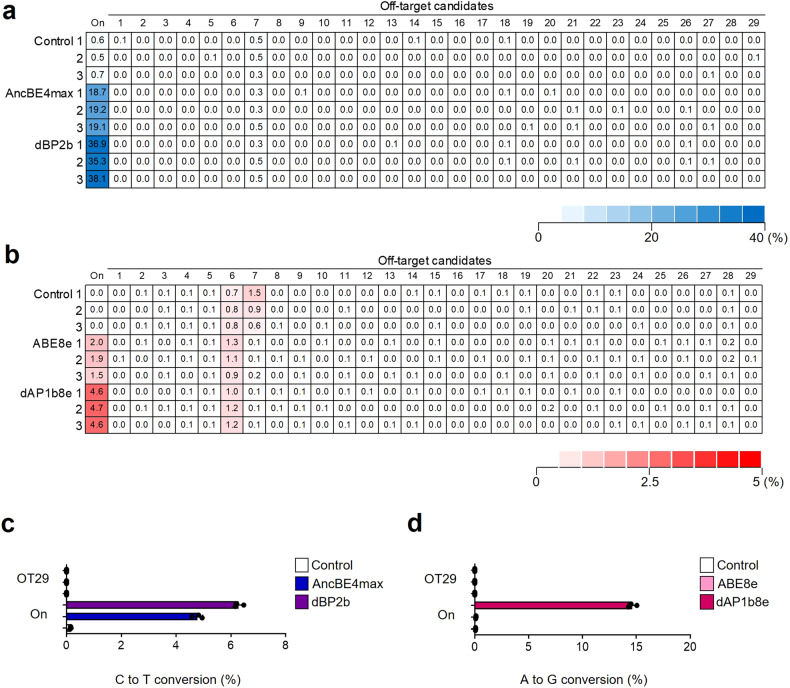
Off-target analyses in myoblasts. **a**, **b** Identification of off-target (OT) effects at the DNA level for CBE and ABE variants in myoblasts on day 10 of transfection. **c**, **d** Off-target analyses at the mRNA level for CBE and ABE variants in myoblasts on day 10 of transfection. Control indicates the untreated group. The data are shown as the mean ± S.D. of three independent replicates. Each dot indicates an individual experiment.

To evaluate the off-target effects of dBP2b and dAP1b8e on the DNA and mRNA levels, we identified 29 potential off-target candidates (OT1-OT29) by the sgRNA of the *Dmd* target with up to three nucleotide mismatches in the mouse genome using Cas9-OFFinder (www.rgenome.net) (Fig. 7a–d; Supplementary Table 5). We then evaluated the off-target effects of dBP2b and dABE8e at both the DNA and mRNA levels. We found that dBP2b did not induce any off-target effects related to C-to-T conversions and indels at the off-target candidate sites at the DNA level. One of the potential off-target sites, OT29, was present in the exon of the *Gpm6b* gene. We performed targeted deep sequencing after cDNA synthesis to verify the off-target effects at the mRNA level, and dBP2b showed no off-target effects at the mRNA level (Fig. 7a, c; Supplementary Fig. 7).

dAP1b8e showed no off-target effects at the mRNA level for OT29 present in the exon of the *Gpm6b* gene among the potential off-target candidate sites (Fig. 7d). However, at the DNA level, A-to-G conversions and indel mutations occurred at two off-target sites (OT5 and OT6) located in the intergenic regions with a low efficiency of <1% (Fig. 7b; Supplementary Fig. 8). In summary, our results indicate that dBP2b and dAP1b8e can achieve precise base editing without indels at the desired target sites and can induce fewer off-target effects than other existing base editors even when expressed over a long time, increasing their feasibility for clinical applications.

## Discussion

Approximately 33,000 known human pathogenic mutations can arise from SNPs^43^. The CRISPR system can use donor DNA to KI sequences of interest through HDR, but these applications are limited owing to their low efficiencies. To overcome these limitations, base editors capable of modifying specific bases have been developed^44^. Hence, a more evolved prime editor consisting of nCas9 and reverse transcriptase was developed. Prime editor is a more accurate genome editing tool that can overcome the low HDR efficiency of the CRISPR system while expanding the editing scope of base editors^41^. However, the role of base editors in clinical applications remains important, as the prime editor has not yet been studied sufficiently in various organisms.

Base editors, such as AncBE4max and ABEmax, have been used to continuously enhance base editing efficiencies through direct evolution. These two improved base editors show higher nucleotide substitution efficiencies for most targets than extant base editors, namely, BE3 and ABE. Moreover, the most recently published ABE8e exhibits a much higher A-to-G conversion efficiency but unfortunately tends to increase the number of indels at the desired target sites.

Herein, we tested whether the base editor variants could remove most indels by using dCas9 instead of nCas9. Using dCas9 in the base editors mostly eliminated the unintended indels at the target sites for both the CBE and ABE variants. However, the nucleotide substitution efficiencies of AncdBE4max and dABEmax tended to be 56.4% and 29.3% lower on average than those of the existing improved base editing methods, AncBE4max and ABEmax. Furthermore, we sought to increase the editing efficiency at the target site using CMPs for the base editors. Among the CMP variants of CBE and ABE, dBP2b showed a 3.0-fold higher substitution level than AncdBE4max, and dAP1b8e induced an average of 8.8-fold increased A-to-G conversion. Our proposed strategies for eliminating unintended indels caused by the current CBE and ABE variants and increasing accurate corrections at the nucleotide level are expected to help develop better genome editing schemes for use in clinical and biological research. In addition, our results indicate the increased safety of base editors and contribute to the advancement of clinical research.

Previous studies have demonstrated that CBE and ABE systems can lead to unwanted RNA editing during genome editing^45–47^. To overcome this, other research teams also introduced evolved base editor variants, such as SECURE-base editor and ABEmaxAW, that have low RNA off-target effects through protein engineering^46,48^. To evaluate the off-target effects at the RNA level, we verified that there were no off-target effects at the mRNA level among the potential off-target candidate sites. However, to apply the base editor variants developed in this study to clinical treatment, it is necessary to verify their safety through more in-depth studies on off-target effects at the DNA and mRNA levels.

Treatment of human pathogenic SNPs requires base editors to have the ability to exchange single nucleotides. Indeed, the success of developing therapeutics using CRISPR/Cas-system-based gene editing in clinical trials depends on how only the desired mutations can be accurately and efficiently corrected without any unintended mutations or off-target effects.

In this study, dCas9, CMP or both were applied to AncBE4max and ABE8e, which showed the greatest improvements by base editing, to identify their editing efficiencies as well as the presence or absence of unwanted mutations. Both AncBE4max and ABE8e showed high editing efficiencies. However, it was confirmed that many unwanted mutations occurred in the target sequences. Accordingly, we have shown AncdBE4max and dABE8e with dCas9 introduction; here, although the unwanted indel mutations were eliminated, the efficiencies were greatly reduced. Therefore, we improved dBP2b and dAP1b8e by applying both dCas9 and CMP, for which the editing efficiencies improved without unwanted indels. Considering the observations in this study together, we recommend using dBP2b and dAP1b8e base editing variants with dCas9 and CMP introductions for successfully eliminating unintended indel mutations while achieving high base editing efficiencies at the target sites. We also showed that dBP2b and dAP1b8e used in primary myoblast cells derived from wild-type or *Dmd* Q871* KO mice had higher base editing efficiencies without any indels and lower off-target effects than conventional base editors. These results offer opportunities for safer and more accurate base editing in disease modeling or clinical gene therapy.

### Supplementary information

Supplementary Information

## Data Availability

The deep-sequencing data are available in the NCBI Sequence Read Archive (SRA) under accession number SRP470076.

## References

[1] Jinek M (2012). A programmable dual-RNA–guided DNA endonuclease in adaptive bacterial immunity. Science.

[2] Rouet P, Smih F, Jasin M (1994). Introduction of double-strand breaks into the genome of mouse cells by expression of a rare-cutting endonuclease. Mol. Cell. Biol..

[3] Chapman JR, Taylor MR, Boulton SJ (2012). Playing the end game: DNA double-strand break repair pathway choice. Mol. Cell.

[4] Doudna JA, Charpentier E (2014). Genome editing. The new frontier of genome engineering with CRISPR-Cas9. Science.

[5] Hsu PD, Lander ES, Zhang F (2014). Development and applications of CRISPR-Cas9 for genome engineering. Cell.

[6] Sander JD, Joung JK (2014). CRISPR-Cas systems for editing, regulating and targeting genomes. Nat. Biotechnol..

[7] Gallagher DN, Haber JE (2018). Repair of a site-specific DNA cleavage: old-school lessons for Cas9-mediated gene editing. ACS Chem. Biol..

[8] Rothkamm K, Kruger I, Thompson LH, Lobrich M (2003). Pathways of DNA double-strand break repair during the mammalian cell cycle. Mol. Cell Biol..

[9] Ciccia A, Elledge SJ (2010). The DNA damage response: making it safe to play with knives. Mol. Cell.

[10] Heyer WD, Ehmsen KT, Liu J (2010). Regulation of homologous recombination in eukaryotes. Annu Rev. Genet..

[11] Iyama T, Wilson DM (2013). DNA repair mechanisms in dividing and non-dividing cells. DNA Repair.

[12] Lin S, Staahl BT, Alla RK, Doudna JA (2014). Enhanced homology-directed human genome engineering by controlled timing of CRISPR/Cas9 delivery. Elife.

[13] Song F, Stieger K (2017). Optimizing the DNA donor template for homology-directed repair of double-strand breaks. Mol. Ther. Nucleic Acids.

[14] Bollen Y, Post J, Koo B-K, Snippert HJ (2018). How to create state-of-the-art genetic model systems: strategies for optimal CRISPR-mediated genome editing. Nucleic Acids Res..

[15] Komor AC, Kim YB, Packer MS, Zuris JA, Liu DR (2016). Programmable editing of a target base in genomic DNA without double-stranded DNA cleavage. Nature.

[16] Gaudelli NM (2017). Programmable base editing of A*T to G*C in genomic DNA without DNA cleavage. Nature.

[17] Marraffini LA, Sontheimer EJ (2010). CRISPR interference: RNA-directed adaptive immunity in bacteria and archaea. Nat. Rev. Genet..

[18] Kim YB (2017). Increasing the genome-targeting scope and precision of base editing with engineered Cas9-cytidine deaminase fusions. Nat. Biotechnol..

[19] Landrum MJ (2014). ClinVar: public archive of relationships among sequence variation and human phenotype. Nucleic Acids Res..

[20] Landrum MJ (2016). ClinVar: public archive of interpretations of clinically relevant variants. Nucleic Acids Res..

[21] Koblan LW (2018). Improving cytidine and adenine base editors by expression optimization and ancestral reconstruction. Nat. Biotechnol..

[22] Grünewald J (2020). A dual-deaminase CRISPR base editor enables concurrent adenine and cytosine editing. Nat. Biotechnol..

[23] Sakata RC (2020). Base editors for simultaneous introduction of C-to-T and A-to-G mutations. Nat. Biotechnol..

[24] Zhang X (2020). Dual base editor catalyzes both cytosine and adenine base conversions in human cells. Nat. Biotechnol..

[25] Nishimasu H (2018). Engineered CRISPR-Cas9 nuclease with expanded targeting space. Science.

[26] Walton RT, Christie KA, Whittaker MN, Kleinstiver BP (2020). Unconstrained genome targeting with near-PAMless engineered CRISPR-Cas9 variants. Science.

[27] Chen L (2021). Programmable C: G to G: C genome editing with CRISPR-Cas9-directed base excision repair proteins. Nat. Commun..

[28] Sun, N. et al. Reconstructed glycosylase base editors GBE2. 0 with enhanced C-to-G base editing efficiency and purity. *Mol. Therapy* **30**, 2452–2463 (2022). 10.1016/j.ymthe.2022.03.023 PMC9263226 35381364

[29] Liang Y (2022). AGBE: a dual deaminase-mediated base editor by fusing CGBE with ABE for creating a saturated mutant population with multiple editing patterns. Nucleic Acids Res..

[30] Kim K (2017). Highly efficient RNA-guided base editing in mouse embryos. Nat. Biotechnol..

[31] Richter MF (2020). Phage-assisted evolution of an adenine base editor with improved Cas domain compatibility and activity. Nat. Biotechnol..

[32] Rothgangl T (2021). In vivo adenine base editing of PCSK9 in macaques reduces LDL cholesterol levels. Nat. Biotechnol..

[33] Kim K (2017). Genome surgery using Cas9 ribonucleoproteins for the treatment of age-related macular degeneration. Genome Res..

[34] Kodaka Y, Asakura Y, Asakura A (2017). Spin infection enables efficient gene delivery to muscle stem cells. Biotechniques.

[35] Bois PR, Grosveld GC (2003). FKHR (FOXO1a) is required for myotube fusion of primary mouse myoblasts. EMBO J..

[36] Bae S, Park J, Kim JS (2014). Cas-OFFinder: a fast and versatile algorithm that searches for potential off-target sites of Cas9 RNA-guided endonucleases. Bioinformatics.

[37] Ryu SM (2018). Adenine base editing in mouse embryos and an adult mouse model of Duchenne muscular dystrophy. Nat. Biotechnol..

[38] Nickoloff JA, Taylor L, Sharma N, Kato TA (2021). Exploiting DNA repair pathways for tumor sensitization, mitigation of resistance, and normal tissue protection in radiotherapy. Cancer Drug Resist..

[39] Wolter F, Schindele P, Beying N, Scheben A, Puchta H (2021). Different DNA repair pathways are involved in single-strand break-induced genomic changes in plants. Plant Cell.

[40] Nambiar TS, Baudrier L, Billon P, Ciccia A (2022). CRISPR-based genome editing through the lens of DNA repair. Mol. Cell.

[41] Ding X (2019). Improving CRISPR-Cas9 genome editing efficiency by fusion with chromatin-modulating peptides. CRISPR J..

[42] Park S-J (2021). Targeted mutagenesis in mouse cells and embryos using an enhanced prime editor. Genome Biol..

[43] Anzalone AV (2019). Search-and-replace genome editing without double-strand breaks or donor DNA. Nature.

[44] Chen, L. et al. Adenine transversion editors enable precise, efficient A*T-to-C*G base editing in mammalian cells and embryos. *Nat. Biotechnol.*10.1038/s41587-023-01821-9 (2023). 10.1038/s41587-023-01821-9 37322276

[45] Grunewald J (2019). Transcriptome-wide off-target RNA editing induced by CRISPR-guided DNA base editors. Nature.

[46] Grunewald J (2019). CRISPR DNA base editors with reduced RNA off-target and self-editing activities. Nat. Biotechnol..

[47] Zhou C (2019). Off-target RNA mutation induced by DNA base editing and its elimination by mutagenesis. Nature.

[48] Rees HA, Wilson C, Doman JL, Liu DR (2019). Analysis and minimization of cellular RNA editing by DNA adenine base editors. Sci. Adv..

