# Supplementary material for: Precise base editing without unintended indels in human cells and mouse primary myoblasts
Source: Exp Mol Med. 2023 Dec 1;55(12):2586–95. doi: 10.1038/s12276-023-01128-4 (PMC10766602; doi:10.1038/s12276-023-01128-4)
Supplement: Supplementary file 1 — Supplementary Information [file 12276_2023_1128_MOESM1_ESM.docx]

**Supplementary Information:**

**Precise Base Editing Without Unintended Indels in Human Cells and Mouse Primary Myoblasts**

**Additional file 1:**

Supplementary Figure S1. dCas9-based CBE and ABE variants reconstituted by introduction of CMPs.

Supplementary Figure S2. Evaluation of A-to-G conversion efficiencies of CMP-conjugated nCas9-based ABEs.

Supplementary Figure S3. Structures of ABE8e conjugated with CMPs.

Supplementary Figure S4. A-to-G conversions and indel frequencies of dCas9- and CMP-introduced ABE8e variant (dAP1b8e).

Supplementary Figure S5. Characteristics of primary myoblasts from wild-type or *Dmd* Q871* KO neonatal skeletal muscles.

Supplementary Figure S6. Identification of green fluorescence protein (GFP) plasmid expressions after transfection in myoblasts.

Supplementary Figure S7. Off-target effects of dBP2b.

Supplementary Figure S8. Off-target effects of dAP1b8e.

Table S1. Target sequences in the human and mouse genome.

Table S2. List of 1^st^ PCR primer for NGS analysis.

Table S3. List of 2^nd^ PCR primer for NGS analysis

Table S4. qPCR Primers of *Dmd* and *Myh3*.

Table S5. List of potential off-target site for *Dmd* target sequence.

Table S6. List of 1^st^ PCR primer for analysis of *Dmd* off-target effects.

Table S7. List of 2^nd^ PCR primer for analysis of *Dmd* off-target effects.

**Supplementary Figures**


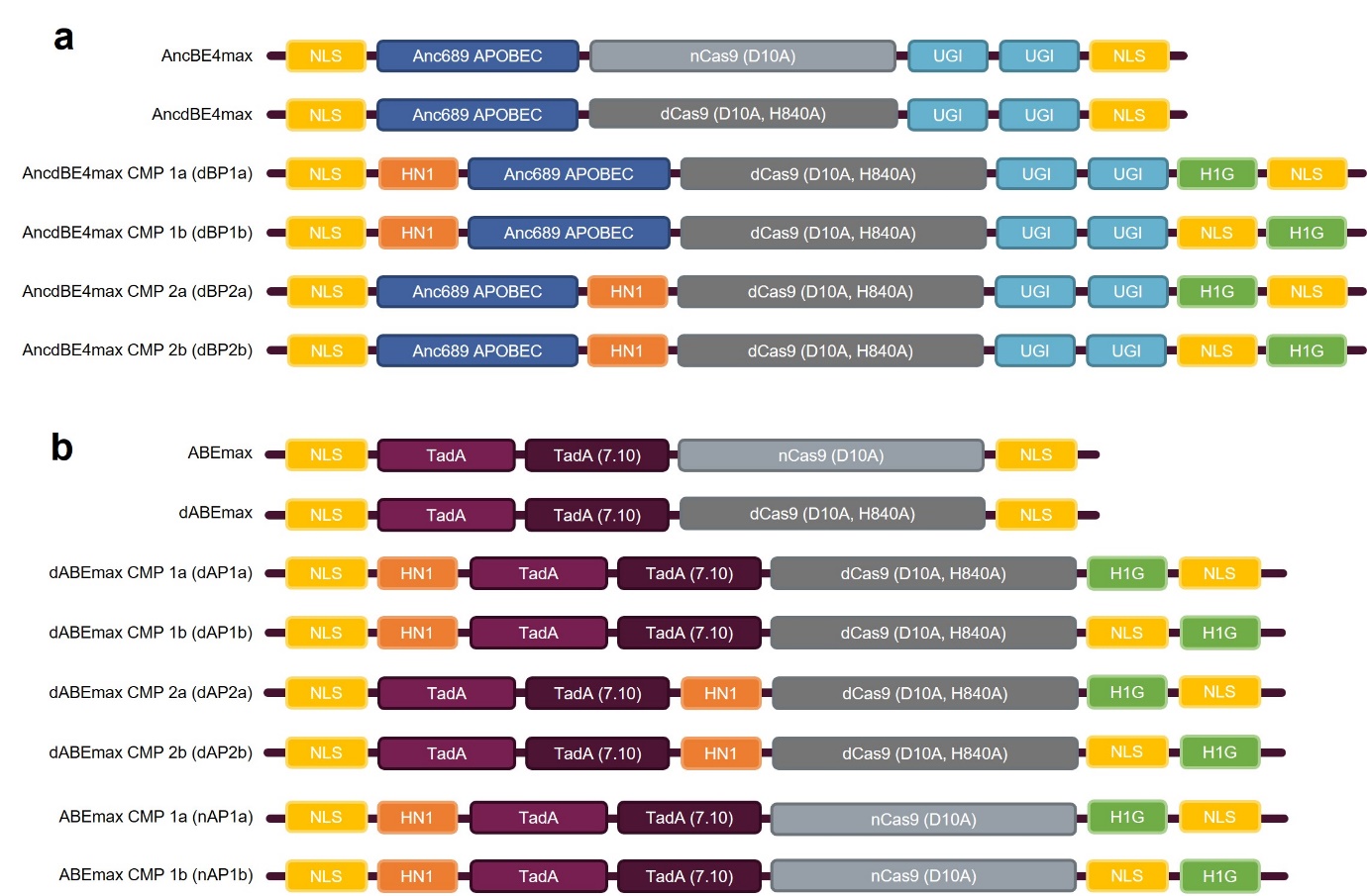


**Supplementary Figure S1. dCas9-based CBE and ABE variants reconstituted by introduction of CMPs.** NLS, nuclear localization sequence; UGI, uracil glycosylase inhibitor; HN1 (HMGN1), high-mobility group nucleosome-binding domain 1; H1G, histone H1 central globular domain.

**
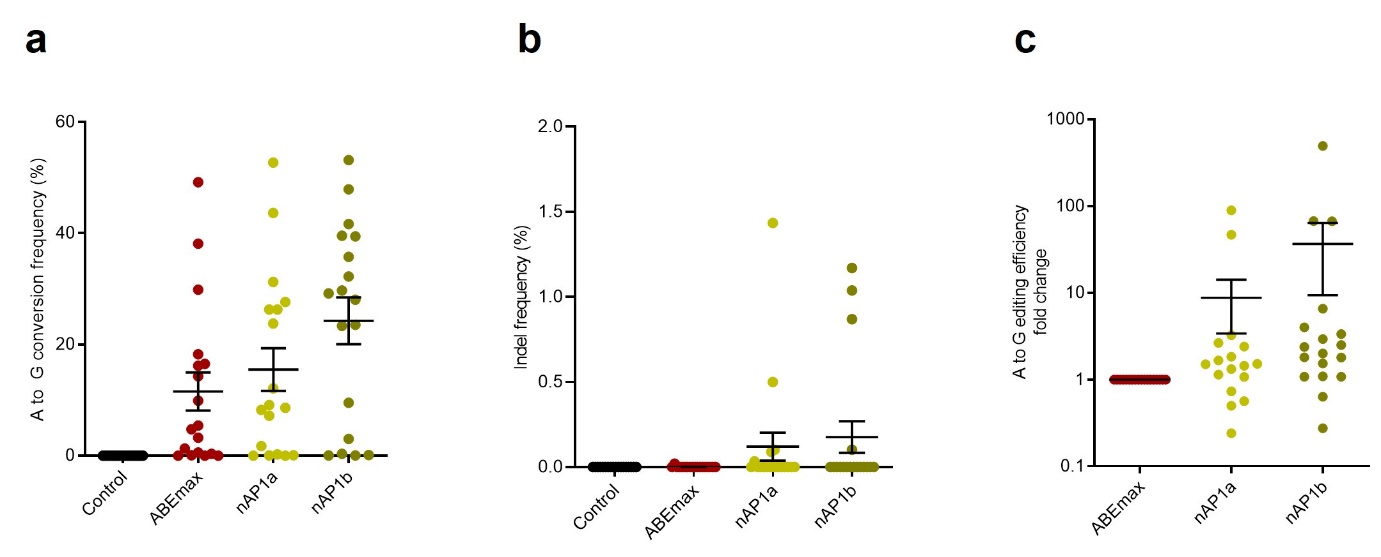
**

**Supplementary Figure S2. Evaluation of A-to-G conversion efficiencies of CMP-conjugated nCas9-based ABEs. a, b** Comparisons of base editing and indel frequencies induced by nAP1a and nAP1b at 18 target sites in HEK293T cells. **c** Fold changes of A-to-G conversions of nAP1a and nAP1b at each target. Control means untreated group. Each dot represents an individual target experiment and is shown together with the mean value.

**
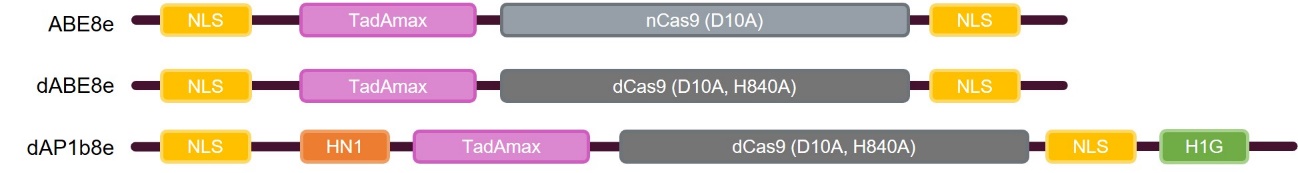
**

**Supplementary Figure S3. Structures of ABE8e conjugated with CMPs.**

**
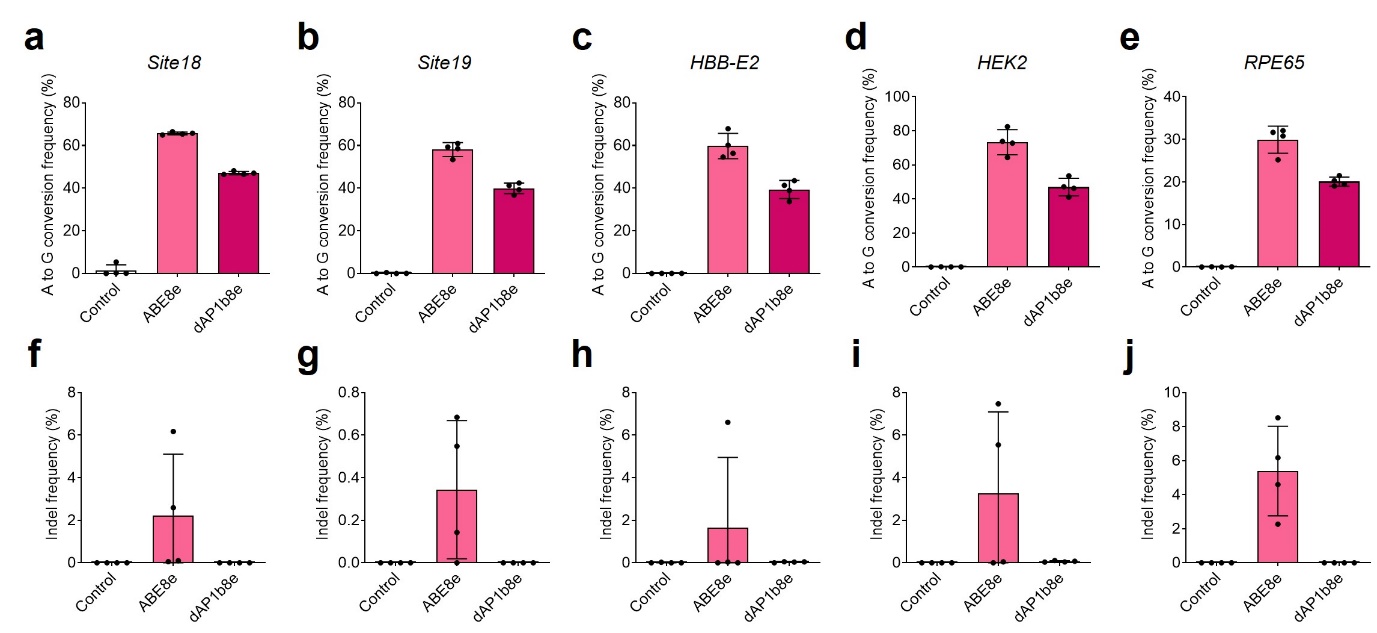
**

**Supplementary Figure S4. A-to-G conversions and indel frequencies of dCas9- and CMP-introduced ABE8e variant (dAP1b8e). a-j** A-to-G substitutions (**a-e**) and indel (**f-j**) frequencies were compared at five different targets using ABE8e and dAP1b8e. dAP1b8e completely removes the indels in all targets but also lowers the frequency of A-to-G conversion. Control means untreated group. The data are shown as mean ± S.D. of four independent experiments. Each dot represents an individual target experiment.

**
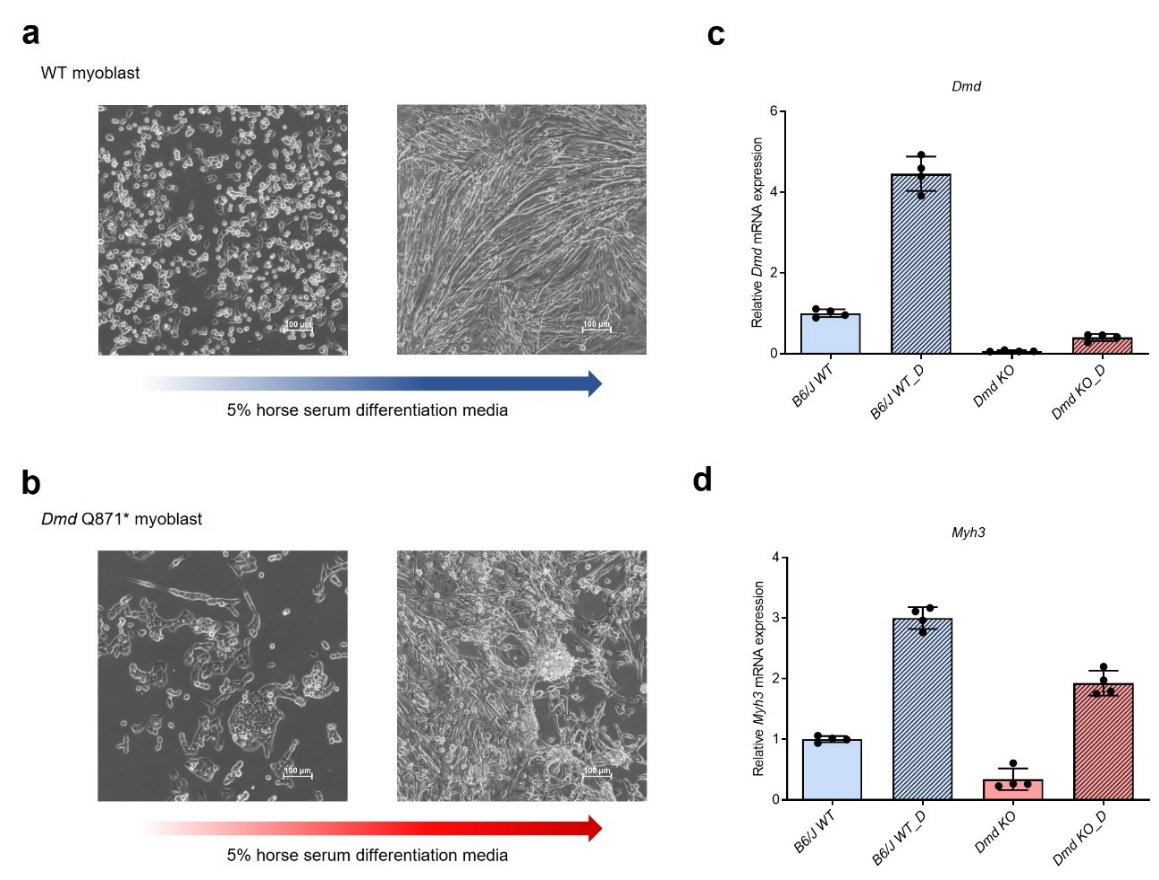
**

**Supplementary Figure S5. Characteristics of primary myoblasts from wild-type or *Dmd* Q871* KO neonatal skeletal muscles. a, b** Primary myoblasts and differentiated cells from wild-type (WT) and *Dmd* KO (*Dmd* Q871*) mice using differentiation media with 5% horse serum. During differentiation, the primary myoblasts fuse with other myoblast cells and elongate. The WT myoblasts form regular veining while differentiating, whereas the *Dmd* Q871* KO myoblasts agglomerate to form a cluster with relatively inconsistent orientation. **c, d** Identification of mRNA expression levels of *Dmd* and *Myh3* as markers of differentiated cells. The mRNA expressions of *Dmd* and *Myh3* were higher in differentiated cells; *Dmd* Q871* KO myoblasts showed less expression of *Dmd* or *Myh3* both before and after differentiation compared to WT. B6/J WT, myoblasts from wild-type C57BL/6-J mouse; B6/J WT_D, differentiated myoblasts from wild-type C57BL/6-J mouse; *Dmd* KO, myoblasts from *Dmd* Q871* knockout C57BL/6-J mouse; *Dmd* KO_D, differentiated myoblasts from *Dmd* Q871* knockout C57BL/6-J mouse. The data are shown as mean ± S.D. of four independent experiments. Each dot represents an individual target experiment.

**
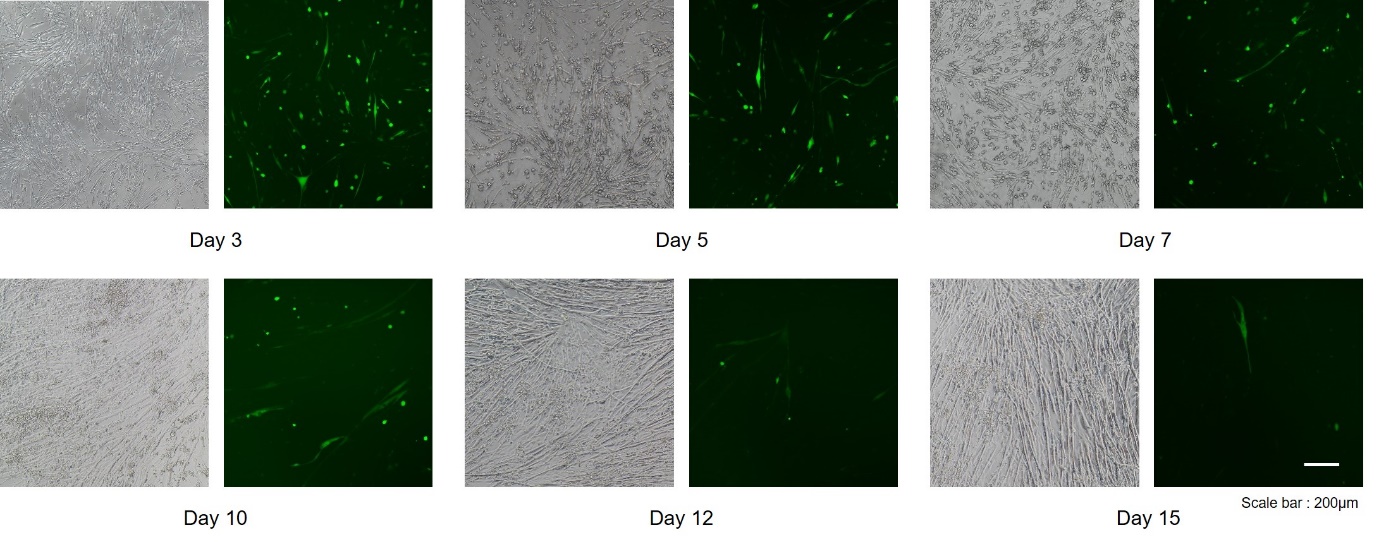
**

**Supplementary Figure S6. Identification of green fluorescence protein (GFP) plasmid expressions after transfection in myoblasts.** Identification of GFP plasmid expressions up to 10 days after transfection. The transfection method is described in the Materials and Methods section.

**
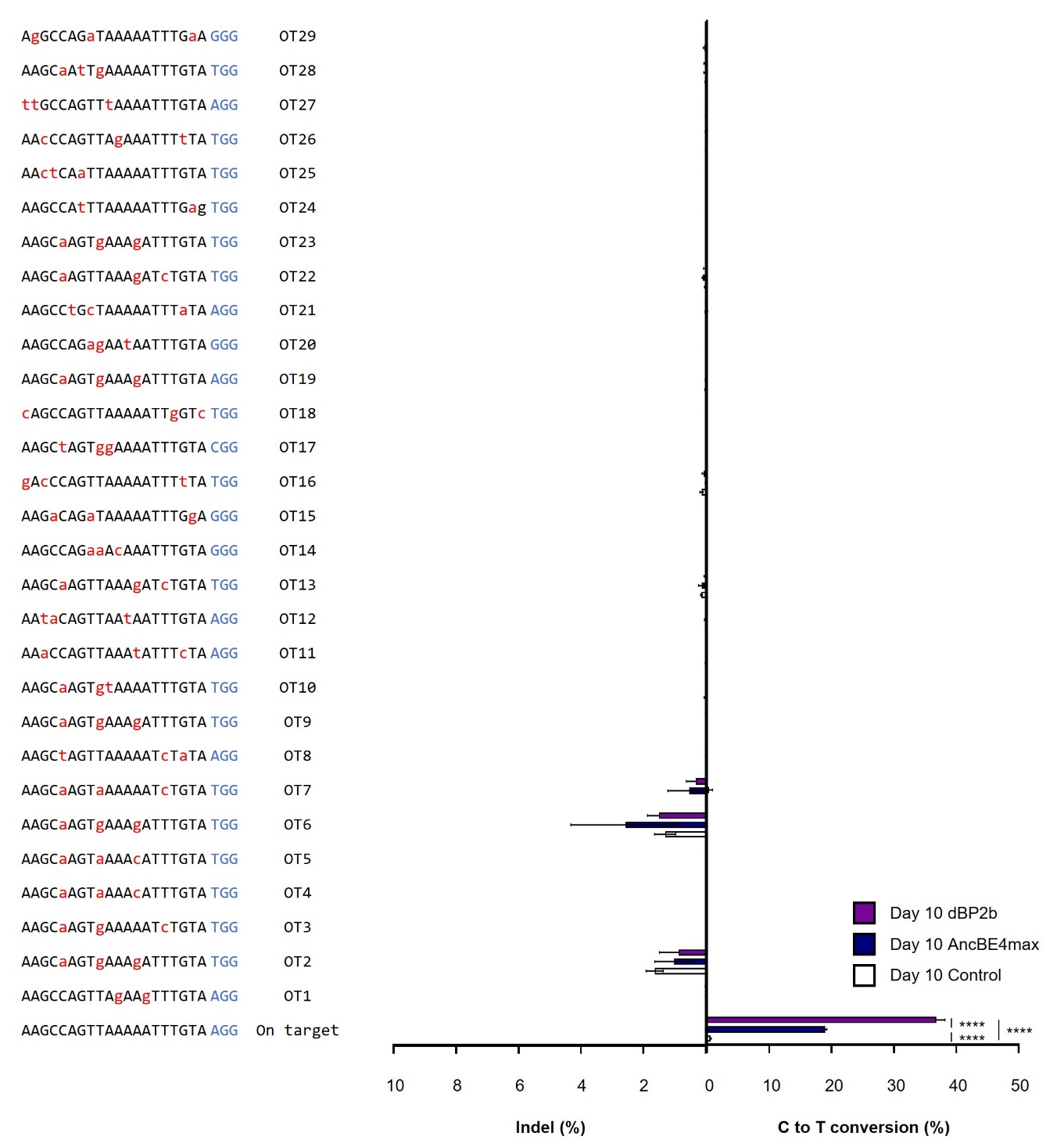
**

**Supplementary Figure S7. Off-target effects of dBP2b.** Twenty-nine off-target candidates were analyzed by targeted deep sequencing. Control means untreated group. The data are shown as mean ± S.D. of four independent experiments.


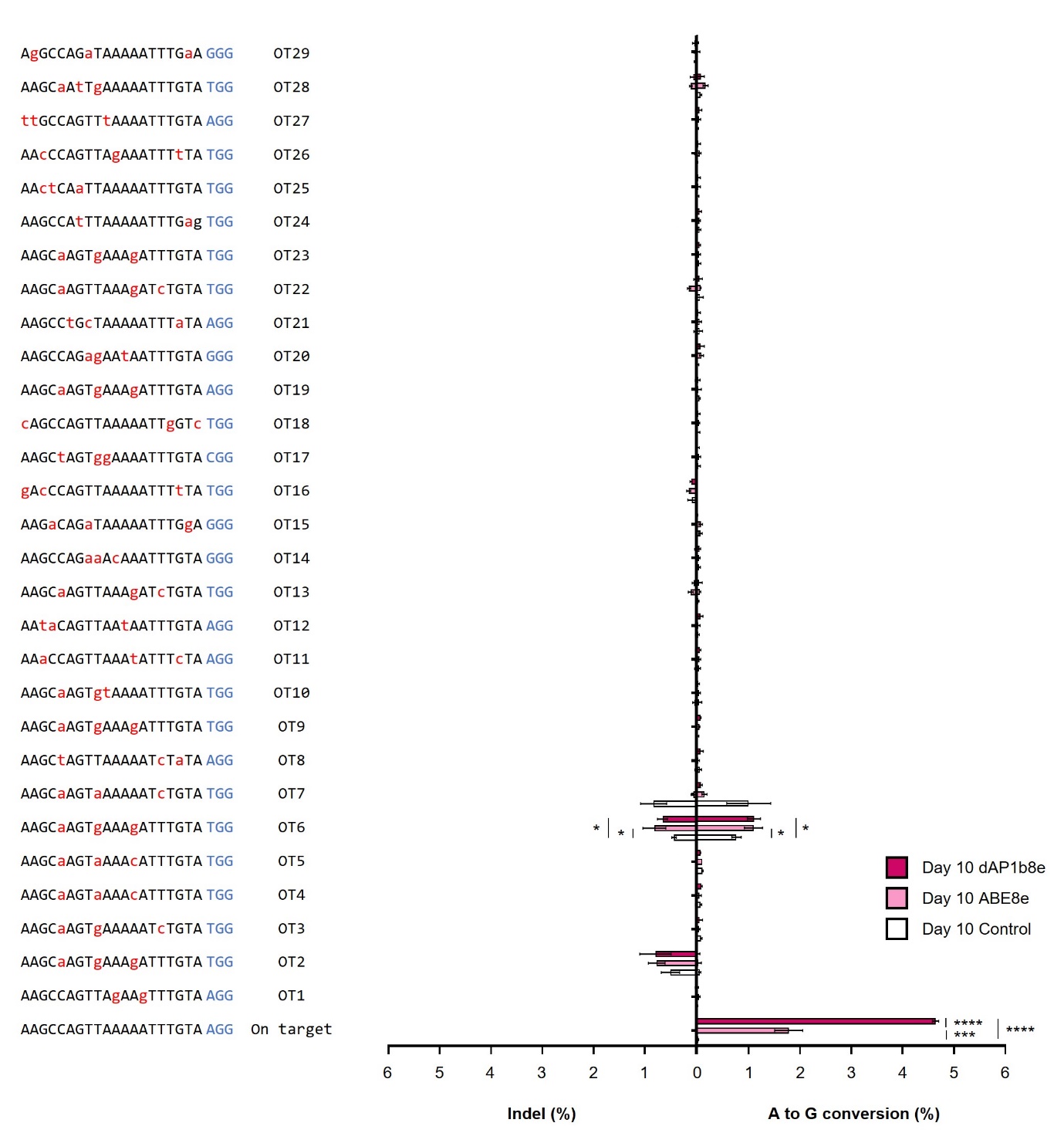


**Supplementary Figure S8. Off-target effects of dAP1b8e.** Twenty-nine off-target candidates were analyzed by targeted deep sequencing. Control means untreated group. The data are shown as mean ± S.D. of four independent experiments.

**Table S1. Target sequences in the human and mouse genome.**

| Name | Sequence (5'-3') |
| --- | --- |
| *HBB* | CTTGCCCCACAGGGCAGTAA |
| *Site18* | ACACACACACTTAGAATCTG |
| *RNF2* | GTCATCTTAGTCATTACCTG |
| *HEK3* | GGCCCAGACTGAGCACGTGA |
| *Site19* | CACACACACTTAGAATCTGT |
| *HBB-E2* | TCAGAAAGTGGTGGCTGGTG |
| *HEK2* | GAACACAAAGCATAGACTGC |
| *EMX1* | GAGTCCGAGCAGAAGAAGAA |
| *FANCF* | GGAATCCCTTCTGCAGCACC |
| *HFE* | ACGTGCCAGGTGGAGCACCC |
| *CXCR4* | CGCGCCAAGTGATAAACACG |
| *JAK2* | CTGCCTTACGATGACAGAAA |
| *ATF1* | TAGGAATCAAACACTTTTAT |
| *BACH2* | ACACTCACTGACCTGTCACA |
| *CCR5* | TGACATCAATTATTATACAT |
| *CREB1* | GCCACAAATCAGATTAATTT |
| *EIF3D* | AGACGACCCTGTCATCCGCA |
| *NEUROG2* | AGGTGCATAGCGGTGCTTGT |
| *POU5F1* | AAATAGCACTTCTGTCATGC |
| *PLXNB2* | GGGCCCAACCTAGGGCATGG |
| *PSMD1* | ACCAGAGCCACAATAAGCCA |
| *TGM2* | TCCTCCACAGCATCTCTTAG |
| *ADAMTS4* | TGGCACCATCAATGGAGATC |
| *MYOCD* | ACTTCAACAAAGAAGGACCC |
| *PRKRA* | AAGAACCAGCTTAATCCTAT |
| *ARG1* | AGGCCCTACAGTATTGAGAA |
| *TTN* | TGAAACTACAGAGCCAGTGA |
| *LGALS3* | CATGATGCGTTATCTGGGTC |
| *AAVS1* | AGGACTTGTCCAAGGAGCGC |
| *DNMT1* | TTCTGTCAGTTGGGACTGTG |
| *Dmd* | AAGCCAGTTAAAAATTTGTA |
| *RPE65* | GATGCCTTGGAAGAAGATGA |
| *Tyr* | CCATAACAGAGACTCTTACA |
| *Lep* | CCAGCAGATGGAGGAGGTCT |
| *Plin1* | TGCCTATGAGAAGGGTGTAC |
| *Prkaa2* | ACAGGCATATGGTTGTCCAT |
| *Prnp* | GGTGGAACACCGGTGGAAGC |
| *Gata3* | GTCACCGCCATGGGTTAGAG |
| *Gjb2* | ACTTCCCCATCTCTCACATC |
| *Kcnq4* | CCTATGCCGACTCGCTCTGG |

**Table S2. List of 1^st^ PCR primer for NGS analysis.**

| Name | | Sequence (5'-3') |
| --- | --- | --- |
| *HBB* | 1st F | ACTGTGTTCACTAGCAACCTC |
| *HBB* | 1st R | TGATGCAATCATTCGTCTGTTTC |
| *Site18, 19* | 1st F | AGAAACACCTTGGAGGAAGTG |
| *Site18, 19* | 1st R | CCAGTTAAGGAGAGGAATGGAAA |
| *RNF2* | 1st F | TGTCAGAACATGCTGGAAGG |
| *RNF2* | 1st R | AGGACTTGCCCAACTTTCTAC |
| *HEK3* | 1st F | TGGGTCACAGTGGCAAAT |
| *HEK3* | 1st R | GGGTAATCTGGTTGATCTCTGAT |
| *HBB-E2* | 1st F | CTCTTTCTTTCAGGGCAATAATGATAC |
| *HBB-E2* | 1st R | GGCAGAATCCAGATGCTCAA |
| *HEK2* | 1st F | TCTAGAGGTCCTAAACCAGTGT |
| *HEK2* | 1st R | CCTCAGCATTCAGCCACTAATA |
| *EMX1* | 1st F | CAGCTCTGTGACCCTTTGTT |
| *EMX1* | 1st R | TGCTTGTCCCTCTGTCAATG |
| *FANCF* | 1st F | TCCAAAGCGAAAGGAAGCG |
| *FANCF* | 1st R | CCAAAGCCGCCCTCTTG |
| *HFE* | 1st F | CTCATCCTTCCTCTTTCCTGTC |
| *HFE* | 1st R | GAACCCTGCCTCTTCCTTAAT |
| *CXCR4* | 1st F | GGCGCGCAATTCAAAGAC |
| *CXCR4* | 1st R | TACAAACCATTCTGGGCTTCA |
| *JAK2* | 1st F | CCCAGAGTAGCTAGGACTACAA |
| *JAK2* | 1st R | ACACGCCAGCCATACAAG |
| *ATF1* | 1st F | CTTGACAGAGGTGTGGGTTATAC |
| *ATF1* | 1st R | CGTAGCTTCCTTGAGGTCATTT |
| *BACH2* | 1st F | CCTAAAGGAGATACCTCAAGGAATAG |
| *BACH2* | 1st R | CAATGATAACTCTCCAGCTCCTT |
| *CCR5* | 1st F | AGAGCCAAGCTCTCCATCTA |
| *CCR5* | 1st R | GAAGATTCCAGAGAAGAAGCCTATAA |
| *CREB1* | 1st F | AGGTGATCTCCCTGCCTT |
| *CREB1* | 1st R | TTCATGATTCCTGGCGTTGA |
| *EIF3D* | 1st F | GTTGTGCTTGCTGTGTTCTATG |
| *EIF3D* | 1st R | GCTTCAGATGCTTTCACCTTTG |
| *NEUROG2* | 1st F | CTTCGCCCACAACTACATCT |
| *NEUROG2* | 1st R | GCTCCATCACACCTTCAGTAA |
| *POU5F1* | 1st F | CCTCCCAAAGTGAAGGGATTAC |
| *POU5F1* | 1st R | AGCATCATGTCTCAGAAGCTAAA |
| *PLXNB2* | 1st F | GTGCTGCCTTGGGACATA |
| *PLXNB2* | 1st R | TAGGGCAGCCTGTCTGA |
| *PSMD1* | 1st F | GAAGCAGGGCAGGCATA |
| *PSMD1* | 1st R | GAATGGCTTTAAGGACTTCCATTT |
| *TGM2* | 1st F | TCTCTCAGGAGGACTCTTTAGTT |
| *TGM2* | 1st R | TCCACTTTGATGTGTGTCTCTT |
| *ADAMTS4* | 1st F | GGAGATCGTGTTTCCAGAGAAG |
| *ADAMTS4* | 1st R | GAACGGCCAGAAGTGTAAGT |
| *MYOCD* | 1st F | CAGAGTGTAGAGAGAGAGAGCA |
| *MYOCD* | 1st R | CCCTGAGAACAAATCCACTACA |
| *PRKRA* | 1st F | GATAGGCTTCTTGGAGGAAGTG |
| *PRKRA* | 1st R | CTGAGGGCCTGTTGATCTTAAA |
| *ARG1* | 1st F | GAACATAGGCTCTGCCACTTAT |
| *ARG1* | 1st R | CCGTTCTTGCACACACAATTTA |
| *TTN* | 1st F | CTGCCTGCCAGAAACTACA |
| *TTN* | 1st R | TCTGAACTCCAGGACTGACT |
| *LGALS3* | 1st F | GCTATAAGTAGAGGAGCGCTAAC |
| *LGALS3* | 1st R | GAAGGAATGCCATCTCACCA |
| *AAVS1* | 1st F | CCTCTAGCAGCAAACACAGA |
| *AAVS1* | 1st R | AGGACGACTCTGGGAACTAT |
| *DNMT1* | 1st F | GCAAGCTTTGGGTTCGTTTC |
| *DNMT1* | 1st R | GGCTCACAGGCACAGATTTA |
| *Dmd* | 1st F | GCTAGAGTATCAAACCAACATCATTAC |
| *Dmd* | 1st R | TGCTTCCTATCTCACCCATCT |
| *RPE65* | 1st F | AGTATTGTGGGAAACCTTACACAT |
| *RPE65* | 1st R | GAGCAGACATTGATGCTCCA |
| *Tyr* | 1st F | CCTCACACTACTTCTGATGAATGA |
| *Tyr* | 1st R | CCCACATTGCATTCCATAGTTC |
| *Lep* | 1st F | TGGCTCGGAATGAACAGAAA |
| *Lep* | 1st R | CTTTGGATGGGTGGTCTACAG |
| *Plin1* | 1st F | AATAGCTCGTGAGAAGGTTGAG |
| *Plin1* | 1st R | TCAGGATGGTAGAAGATGGTAGA |
| *Prkaa2* | 1st F | CTGGTTTGCTTTGTCTGACTTG |
| *Prkaa2* | 1st R | AGGCATGTTGAGTGGATCTG |
| *Prnp* | 1st F | GATGGGATGAGCTGTGTGTT |
| *Prnp* | 1st R | GTTAGGGTAGCGGTACATGTTT |
| *Gata3* | 1st F | ATCCCTGAGCCACATCTCT |
| *Gata3* | 1st R | CCGGATTCAGTGGTTGGAAT |
| *Gjb2* | 1st F | CTCCACCAGCATTGGAAAGA |
| *Gjb2* | 1st R | AAAGATGACCCGGAAGAAGATG |
| *Kcnq4* | 1st F | GCCTGAAAGATGGAGCAAGA |
| *Kcnq4* | 1st R | CCACAGCCTTAGTGTGAAGAG |

**Table S3. List of 2^nd^ PCR primer for NGS analysis.**

| Name | | Sequence (5'-3') |
| --- | --- | --- |
| *HBB* | 2nd F | ACACTCTTTCCCTACACGACGCTCTTCCGATCTCACTAGCAACCTCAAACAGACA |
| *HBB* | 2nd R | GTGACTGGAGTTCAGACGTGTGCTCTTCCGATCTGTGCCTATCAGAAACCCAAGAG |
| *Site18, 19* | 2nd F | ACACTCTTTCCCTACACGACGCTCTTCCGATCTTGCTGTCCAAGAAGCAACA |
| *Site18, 19* | 2nd R | GTGACTGGAGTTCAGACGTGTGCTCTTCCGATCTACCTCTAGTTCTCAAACTTCAGC |
| *RNF2* | 2nd F | ACACTCTTTCCCTACACGACGCTCTTCCGATCTTGCAGACAAACGGAACTCAA |
| *RNF2* | 2nd R | GTGACTGGAGTTCAGACGTGTGCTCTTCCGATCTGCCAACATACAGAAGTCAGGAA |
| *HEK3* | 2nd F | ACACTCTTTCCCTACACGACGCTCTTCCGATCTGTCTATTTCTGCTGCAAGTAAGC |
| *HEK3* | 2nd R | GTGACTGGAGTTCAGACGTGTGCTCTTCCGATCTACCTCTCCAAACTTGTCAACCAGTATCC |
| *HBB-E2* | 2nd F | ACACTCTTTCCCTACACGACGCTCTTCCGATCTACCTCTTATCTTCCTCCCACA |
| *HBB-E2* | 2nd R | GTGACTGGAGTTCAGACGTGTGCTCTTCCGATCTAGTTGGACTTAGGGAACAAAGG |
| *HEK2* | 2nd F | ACACTCTTTCCCTACACGACGCTCTTCCGATCTGGACGTCTGCCCAATATGTAA |
| *HEK2* | 2nd R | GTGACTGGAGTTCAGACGTGTGCTCTTCCGATCTCATCTGTCAAACTGTGCGTATG |
| *EMX1* | 2nd F | ACACTCTTTCCCTACACGACGCTCTTCCGATCTCTGGCCCAGGTGAAGGT |
| *EMX1* | 2nd R | GTGACTGGAGTTCAGACGTGTGCTCTTCCGATCTTCGTGGGTTTGTGGTTGC |
| *FANCF* | 2nd F | ACACTCTTTCCCTACACGACGCTCTTCCGATCTTGCAGAGAGGCGTATCATTTC |
| *FANCF* | 2nd R | GTGACTGGAGTTCAGACGTGTGCTCTTCCGATCTAAAGCGCCGATGGATGT |
| *HFE* | 2nd F | ACACTCTTTCCCTACACGACGCTCTTCCGATCTGCCAAGGAGTTCGAACCTAAA |
| *HFE* | 2nd R | GTGACTGGAGTTCAGACGTGTGCTCTTCCGATCTCATAATTACCTCCTCAGGCACTC |
| *CXCR4* | 2nd F | ACACTCTTTCCCTACACGACGCTCTTCCGATCTCGGGAGAGTGAGGAAATGAAA |
| *CXCR4* | 2nd R | GTGACTGGAGTTCAGACGTGTGCTCTTCCGATCTGTATATTGGGCGGGAGTGTC |
| *JAK2* | 2nd F | ACACTCTTTCCCTACACGACGCTCTTCCGATCTCCCGCAAAGTGCTAGGATTA |
| *JAK2* | 2nd R | GTGACTGGAGTTCAGACGTGTGCTCTTCCGATCTGATACACCTGAAGAACTGGATCTAT |
| *ATF1* | 2nd F | ACACTCTTTCCCTACACGACGCTCTTCCGATCTAAACTCCACTAGGAAATCCAT |
| *ATF1* | 2nd R | GTGACTGGAGTTCAGACGTGTGCTCTTCCGATCTCTCGAGAATGTCGCAGAAAG |
| *BACH2* | 2nd F | ACACTCTTTCCCTACACGACGCTCTTCCGATCTTAACAAGCAGAACGGAGTATGG |
| *BACH2* | 2nd R | GTGACTGGAGTTCAGACGTGTGCTCTTCCGATCTGTCTTAGGATGCAGGGAACTG |
| *CCR5* | 2nd F | ACACTCTTTCCCTACACGACGCTCTTCCGATCTCATTCATGGAGGGCAACTAAATAC |
| *CCR5* | 2nd R | GTGACTGGAGTTCAGACGTGTGCTCTTCCGATCTAAGATGAACACCAGTGAGTAGAG |
| *CREB1* | 2nd F | ACACTCTTTCCCTACACGACGCTCTTCCGATCTAACAGAGTGGCAGTGCTT |
| *CREB1* | 2nd R | GTGACTGGAGTTCAGACGTGTGCTCTTCCGATCTGTAGTTGCTTTCAGGCAGTTT |
| *EIF3D* | 2nd F | ACACTCTTTCCCTACACGACGCTCTTCCGATCTATGTCTTTGCCTGCTCTTCC |
| *EIF3D* | 2nd R | GTGACTGGAGTTCAGACGTGTGCTCTTCCGATCTAAGCCGACAGCATTCCTAAC |
| *NEUROG2* | 2nd F | ACACTCTTTCCCTACACGACGCTCTTCCGATCTCAGGGCGTGGAAAGGAG |
| *NEUROG2* | 2nd R | GTGACTGGAGTTCAGACGTGTGCTCTTCCGATCTCCCTACAGCTGCACTTTATC |
| *POU5F1* | 2nd F | ACACTCTTTCCCTACACGACGCTCTTCCGATCTCAGAGCAGGAGTGGAAGTTTAT |
| *POU5F1* | 2nd R | GTGACTGGAGTTCAGACGTGTGCTCTTCCGATCTTCACTATAGAGGCATCCTAATTGAT |
| *PLXNB2* | 2nd F | ACACTCTTTCCCTACACGACGCTCTTCCGATCTTTCTCCTCCTCCCATCTCG |
| *PLXNB2* | 2nd R | GTGACTGGAGTTCAGACGTGTGCTCTTCCGATCTGTGTTCACTGTCGAGTCTCC |
| *PSMD1* | 2nd F | ACACTCTTTCCCTACACGACGCTCTTCCGATCTCACTTATTGTCCATCTGCAAGC |
| *PSMD1* | 2nd R | GTGACTGGAGTTCAGACGTGTGCTCTTCCGATCTCCACTATGCCTATTAGACTGTAGAAG |
| *TGM2* | 2nd F | ACACTCTTTCCCTACACGACGCTCTTCCGATCTCAAAGCTGGATCCCTGGTAG |
| *TGM2* | 2nd R | GTGACTGGAGTTCAGACGTGTGCTCTTCCGATCTCTCACGGCTACTGCTTCTC |
| *ADAMTS4* | 2nd F | ACACTCTTTCCCTACACGACGCTCTTCCGATCTGGCTGACAGTGCAGTACC |
| *ADAMTS4* | 2nd R | GTGACTGGAGTTCAGACGTGTGCTCTTCCGATCTACTCTTCCGGCGTAGGAT |
| *MYOCD* | 2nd F | ACACTCTTTCCCTACACGACGCTCTTCCGATCTTGTAATGATATTTAGCCCAACTCAC |
| *MYOCD* | 2nd R | GTGACTGGAGTTCAGACGTGTGCTCTTCCGATCTTTGGCTTAAACTAAATGACCTCTG |
| *PRKRA* | 2nd F | ACACTCTTTCCCTACACGACGCTCTTCCGATCTTAGCTTTGCAGTTCCTGACC |
| *PRKRA* | 2nd R | GTGACTGGAGTTCAGACGTGTGCTCTTCCGATCTCTCTGTTAATCTAATGATCGTAAGTTGG |
| *ARG1* | 2nd F | ACACTCTTTCCCTACACGACGCTCTTCCGATCTAAAGGGTTCCTGCTGTGAG |
| *ARG1* | 2nd R | GTGACTGGAGTTCAGACGTGTGCTCTTCCGATCTCCTGCTCTTTCCTTCAGAGTG |
| *TTN* | 2nd F | ACACTCTTTCCCTACACGACGCTCTTCCGATCTTTTGTCGGAGAAGACTCCATTC |
| *TTN* | 2nd R | GTGACTGGAGTTCAGACGTGTGCTCTTCCGATCTCTGTTCACCTTTACCTTTCCTTTC |
| *LGALS3* | 2nd F | ACACTCTTTCCCTACACGACGCTCTTCCGATCTGAATGCTTTCTGTCCCGTAATTG |
| *LGALS3* | 2nd R | GTGACTGGAGTTCAGACGTGTGCTCTTCCGATCTAGGTGCCTGTCCAGGATAA |
| *AAVS1* | 2nd F | ACACTCTTTCCCTACACGACGCTCTTCCGATCTAGGTATAGGGAGGGAGGAGT |
| *AAVS1* | 2nd R | GTGACTGGAGTTCAGACGTGTGCTCTTCCGATCTACCGGTGGGACCTTCTT |
| *DNMT1* | 2nd F | ACACTCTTTCCCTACACGACGCTCTTCCGATCTGCTGGCTCATGTTCGAGAG |
| *DNMT1* | 2nd R | GTGACTGGAGTTCAGACGTGTGCTCTTCCGATCTATGCAGCCTTCACGCTT |
| *Dmd* | 2nd F | ACACTCTTTCCCTACACGACGCTCTTCCGATCTGCTACAACAATTGGAACAGATGAC |
| *Dmd* | 2nd R | GTGACTTGGAGTTCAGACGTGTGCTCTTCCGATCTTCTTCACTGTACAGAGCTCAATG |
| *RPE65* | 2nd F | ACACTCTTTCCCTACACGACGCTCTTCCGATCTACAGCTCTGTAAGCTGAATGT |
| *RPE65* | 2nd R | GTGACTGGAGTTCAGACGTGTGCTCTTCCGATCTTCAACCTTACTCCTTTCCTAACG |
| *Tyr* | 2nd F | ACACTCTTTCCCTACACGACGCTCTTCCGATCTTCCTGACTCTGAGTAACCCTTC |
| *Tyr* | 2nd R | GTGACTGGAGTTCAGACGTGTGCTCTTCCGATCTGACTCTTGGAGGTAGCTGTAGT |
| *Lep* | 2nd F | ACACTCTTTCCCTACACGACGCTCTTCCGATCTCCCATTCTGAGTTTGTCCAAGAT |
| *Lep* | 2nd R | GTGACTGGAGTTCAGACGTGTGCTCTTCCGATCTTTCCAGGACGCCATCCA |
| *Plin1* | 2nd F | ACACTCTTTCCCTACACGACGCTCTTCCGATCTTTCTTTGCTGACAGGAGCAG |
| *Plin1* | 2nd R | GTGACTGGAGTTCAGACGTGTGCTCTTCCGATCTAGTCTATGAGGACTCACACTGG |
| *Prkaa2* | 2nd F | ACACTCTTTCCCTACACGACGCTCTTCCGATCTCCTGCTTGCTAGGTTGAAGA |
| *Prkaa2* | 2nd R | GTGACTGGAGTTCAGACGTGTGCTCTTCCGATCTTTGTGAGGGTGAATGGATACTG |
| *Prnp* | 2nd F | ACACTCTTTCCCTACACGACGCTCTTCCGATCTTGTGACTATGTGGACTGATGTC |
| *Prnp* | 2nd R | GTGACTGGAGTTCAGACGTGTGCTCTTCCGATCTCCAACTACCACCATGAGGTT |
| *Gata3* | 2nd F | ACACTCTTTCCCTACACGACGCTCTTCCGATCTCACTCCAGCCACATGCT |
| *Gata3* | 2nd R | GTGACTGGAGTTCAGACGTGTGCTCTTCCGATCTGTCGCTTTCGGGCTTCA |
| *Gjb2* | 2nd F | ACACTCTTTCCCTACACGACGCTCTTCCGATCTTATGCTACGACCACCACTTC |
| *Gjb2* | 2nd R | GTGACTGGAGTTCAGACGTGTGCTCTTCCGATCTGATACGGACCTTCTGGGTTT |
| *Kcnq4* | 2nd F | ACACTCTTTCCCTACACGACGCTCTTCCGATCTTGATCACCGCCTGGTACAT |
| *Kcnq4* | 2nd R | GTGACTGGAGTTCAGACGTGTGCTCTTCCGATCTTAGGAAGACAGAACCTGGGAAG |

**Table S4. qPCR primers of *Dmd* and *Myh3*.**

| Name | | Sequence (5'-3') |
| --- | --- | --- |
| *Gapdh* | F | GCTGAGTATGTCGTGGAGTCTA |
| *Gapdh* | R | GTGGTTCACACCCATCACAA |
| *Dmd* | F | ATTACTCCACTTGGAGATGAGC |
| *Dmd* | R | ACTGCTCCTCCTGTTTCATTTA |
| *Myh3* | F | TGCCTATGAAGAAGCCTTGG |
| *Myh3* | R | TTACCGTTCTCGGCAATCTG |

**Table S5. List of potential off-target sites for *Dmd* target sequence.**

|  | Target sequence (5'-3') | PAM | Chromosome | Position | Direction | Mismatches | Overlapped gene |
| --- | --- | --- | --- | --- | --- | --- | --- |
| On target | AAGCCAGTTAAAAATTTGTA | AGG | X | 83781748 | + | 0 | Intergenic region |
| OT1 | AAGCCAGTTAgAAgTTTGTA | AGG | X | 60354718 | + | 2 | *Atp11c* intron |
| OT2 | AAGCaAGTgAAAgATTTGTA | TGG | 1 | 7687839 | - | 3 | Intergenic region |
| OT3 | AAGCaAGTgAAAAATcTGTA | TGG | 1 | 114729484 | - | 3 | Intergenic region |
| OT4 | AAGCaAGTaAAAcATTTGTA | TGG | 1 | 139933967 | + | 3 | *Gm16332* intron |
| OT5 | AAGCaAGTaAAAcATTTGTA | TGG | 1 | 140152156 | + | 3 | Intergenic region |
| OT6 | AAGCaAGTgAAAgATTTGTA | TGG | 2 | 16963555 | - | 3 | Intergenic region |
| OT7 | AAGCaAGTaAAAAATcTGTA | TGG | 2 | 108527433 | - | 3 | Intergenic region |
| OT8 | AAGCtAGTTAAAAATcTaTA | AGG | 3 | 16613815 | + | 3 | Intergenic region |
| OT9 | AAGCaAGTgAAAgATTTGTA | TGG | 3 | 92518989 | + | 3 | Intergenic region |
| OT10 | AAGCaAGTgtAAAATTTGTA | TGG | 3 | 123915964 | - | 3 | Intergenic region |
| OT11 | AAaCCAGTTAAAtATTTcTA | AGG | 4 | 72159600 | + | 3 | *Tle1* intron |
| OT12 | AAtaCAGTTAAtAATTTGTA | AGG | 4 | 78045958 | + | 3 | *ptprd* intron |
| OT13 | AAGCaAGTTAAAgATcTGTA | TGG | 6 | 42885010 | - | 3 | Intergenic region |
| OT14 | AAGCCAGaaAcAAATTTGTA | GGG | 6 | 64297703 | - | 3 | *Grid2* intron |
| OT15 | AAGaCAGaTAAAAATTTGgA | GGG | 6 | 86907351 | - | 3 | *Aak1* intron |
| OT16 | gAcCCAGTTAAAAATTTtTA | TGG | 6 | 94052484 | + | 3 | *Magi1* intron |
| OT17 | AAGCtAGTggAAAATTTGTA | CGG | 6 | 147759582 | - | 3 | Intergenic region |
| OT18 | cAGCCAGTTAAAAATTgGTc | TGG | 7 | 88911106 | - | 3 | Intergenic region |
| OT19 | AAGCaAGTgAAAgATTTGTA | AGG | 10 | 15119019 | - | 3 | Intergenic region |
| OT20 | AAGCCAGagAAtAATTTGTA | GGG | 11 | 94052933 | + | 3 | *Spag9* intron |
| OT21 | AAGCCtGcTAAAAATTTaTA | AGG | 12 | 40906354 | + | 3 | Intergenic region |
| OT22 | AAGCaAGTTAAAgATcTGTA | TGG | 13 | 7252373 | - | 3 | Intergenic region |
| OT23 | AAGCaAGTgAAAgATTTGTA | TGG | 13 | 12809865 | - | 3 | Intergenic region |
| OT24 | AAGCCAtTTAAAAATTTGag | TGG | 13 | 47235657 | - | 3 | *Rnf144b* intron |
| OT25 | AActCAaTTAAAAATTTGTA | TGG | 14 | 26539529 | - | 3 | Intergenic region |
| OT26 | AAcCCAGTTAgAAATTTtTA | TGG | 17 | 43052831 | + | 3 | *Tnfrsf21* intron |
| OT27 | ttGCCAGTTtAAAATTTGTA | AGG | 17 | 80462531 | - | 3 | *Sos1* intron |
| OT28 | AAGCaAtTgAAAAATTTGTA | TGG | 17 | 81921018 | + | 3 | Intergenic region |
| OT29 | AgGCCAGaTAAAAATTTGaA | GGG | X | 166385994 | + | 3 | *Gpm6b* exon |

**Table S6. List of 1^st^ PCR primer for analysis of *Dmd* off-target effects.**

| Name | Sequence (5'-3') |
| --- | --- |
| Dmd-OT1 1st F | GTACCTCACGGTGGCAAATA |
| Dmd-OT1 1st R | TGACTCTCTTCAGCAGTTCTTAC |
| Dmd-OT2 1st F | GACAACAAATGGAGAGCAAAGG |
| Dmd-OT2 1st R | GGTGATTCCACCAGAGGTTATATT |
| Dmd-OT3 1st F | AATCGTATACTTCAATGACCCTACA |
| Dmd-OT3 1st R | ACAGAACCAAGACTTCAGCAATA |
| Dmd-OT4 1st F | GTGATACAAATTGGAAAGGAAGAAGTC |
| Dmd-OT4 1st R | GTGATTCCACCAGAGGTTCTTT |
| Dmd-OT5 1st F | ACTGTTCGAAGATGATATGACAGTA |
| Dmd-OT5 1st R | GTGATTCCACCAGAGGTTCTT |
| Dmd-OT6 1st F | TTCCACCAGAGAACTCCTAAAC |
| Dmd-OT6 1st R | TGAGGTCAGAGATGGTGATTTC |
| Dmd-OT7 1st F | CTTGAAGTCCTAGCCAGAGTAATG |
| Dmd-OT7 1st R | CAGCTTGAGGTCAGGGATTATG |
| Dmd-OT8 1st F | GAGCTCTACTCACACCCTTAAT |
| Dmd-OT8 1st R | GCCCAATACCAGTAAGAACTAATC |
| Dmd-OT9 1st F | CTCCCAAGTGCTGGGATTAAA |
| Dmd-OT9 1st R | TGAGATTAGGGCTGGTGACT |
| Dmd-OT10 1st F | TGCCCACTCTCTCCCTATTT |
| Dmd-OT10 1st R | AGGTCAGGGATGGTGATTCT |
| Dmd-OT11 1st F | ACAAAGTGTTGAGACCGAGAG |
| Dmd-OT11 1st R | TTGAGATGAAGGTGGACTACTAAAG |
| Dmd-OT12 1st F | GAAGTCATGTGATGAGGAGATACA |
| Dmd-OT12 1st R | CCAATTTGGGTTGGTCATTGG |
| Dmd-OT13 1st F | AAAGGAGGTCAAAGGGATACAA |
| Dmd-OT13 1st R | AGATCAGGGATGGGCAATTC |
| Dmd-OT14 1st F | AAGGAGGATGAATGGGATGAAG |
| Dmd-OT14 1st R | GTGTGCAATAAACCACAGTGATAG |
| Dmd-OT15 1st F | AGTCGTTAGGCCTGTGAGA |
| Dmd-OT15 1st R | CAATTCTGTAGCTGAGGGTGAC |
| Dmd-OT16 1st F | GCGGTGCCTTACAAGATACA |
| Dmd-OT16 1st R | CTTTCCCAAGCGTCCTCAT |
| Dmd-OT17 1st F | CCACACACTAAGGAGGGAAAG |
| Dmd-OT17 1st R | TCTGGCCCAAACAGAATGTAA |
| Dmd-OT18 1st F | AGACTATGGATCCTGGGAGAG |
| Dmd-OT18 1st R | ACTCAATGTGATCTCTTGACTATACTG |
| Dmd-OT19 1st F | GCAATAAGACATCCATAGAAGATCA |
| Dmd-OT19 1st R | GCTTGAAATCAGGGATGGTG |
| Dmd-OT20 1st F | AGCATGACAGTGGGAGAAAC |
| Dmd-OT20 1st R | TGCACTCAGCTTGGGAAAT |
| Dmd-OT21 1st F | AGACAGCTTTAGGTTCTGTGTAG |
| Dmd-OT21 1st R | AGTCAGAGTCAAGAACCAAGAAA |
| Dmd-OT22 1st F | ACCCTTAAGTGACCCAGAGA |
| Dmd-OT22 1st R | TGTGATTCCCTCAGAAGTTCTTT |
| Dmd-OT23 1st F | AACTGGAAAGGAAGAAGTCAAA |
| Dmd-OT23 1st R | CCCAAACCTATGTGGTACAATAA |
| Dmd-OT24 1st F | GCCTTGACAGCCTGGATATT |
| Dmd-OT24 1st R | CTTCCATGTGCTGTACGTAGG |
| Dmd-OT25 1st F | TTGGCCATCTTACCCTCCT |
| Dmd-OT25 1st R | AGAATTGCTATAACCTGCCAGAG |
| Dmd-OT26 1st F | GAAGAGGGCCATGAGTCAAA |
| Dmd-OT26 1st R | TCTCCCACATCCCTAGACTATAC |
| Dmd-OT27 1st F | CACACAAACACACACAAATAAAGG |
| Dmd-OT27 1st R | CCTGCCAAGTGTGGTATGTA |
| Dmd-OT28 1st F | TTTGGCCAGTGGTCTCTATTC |
| Dmd-OT28 1st R | GTGATTCCACCAGAGGTTCTT |
| Dmd-OT29 1st F | GAGCAGTCAGTATCTCTCAATGG |
| Dmd-OT29 1st R | TAAAGGCCACATTCCCTTCTC |
| Dmd-cDNA On 1st F | GACAAGCTTCAGAACAACTGAAC |
| Dmd-cDNA On 1st R | GCCCTCACACCATCAAAGA |

**Table S7. List of 2^nd^ PCR primer for analysis of *Dmd* off-target effects.**

| Name | Sequence (5'-3') |
| --- | --- |
| Dmd-OT1 2nd F | ACACTCTTTCCCTACACGACGCTCTTCCGATCTCTGCAGCATCAAAGAATTCACA |
| Dmd-OT1 2nd R | GTGACTGGAGTTCAGACGTGTGCTCTTCCGATCTGTCTGCCTTATTCCTCCTCATC |
| Dmd-OT2 2nd F | ACACTCTTTCCCTACACGACGCTCTTCCGATCTGCCTTCTTCTACACAAAGGATAAAC |
| Dmd-OT2 2nd R | GTGACTGGAGTTCAGACGTGTGCTCTTCCGATCTACAATGAGCATGGGAGATCTTT |
| Dmd-OT3 2nd F | ACACTCTTTCCCTACACGACGCTCTTCCGATCTCTCAAACAAATCAGTAGCCTTCTTC |
| Dmd-OT3 2nd R | GTGACTGGAGTTCAGACGTGTGCTCTTCCGATCTGAGATCTTAAGCCTCTAGCTACAAAT |
| Dmd-OT4 2nd F | ACACTCTTTCCCTACACGACGCTCTTCCGATCTCAAAGGATAAGCAGGCTGAGA |
| Dmd-OT4 2nd R | GTGACTGGAGTTCAGACGTGTGCTCTTCCGATCTTGGGAGATCTTTCCAACTTCTG |
| Dmd-OT5 2nd F | ACACTCTTTCCCTACACGACGCTCTTCCGATCTACAAAGGATAAACAGGCTGAGA |
| Dmd-OT5 2nd R | GTGACTGGAGTTCAGACGTGTGCTCTTCCGATCTATCCATGAGCATGGGATATCTTT |
| Dmd-OT6 2nd F | ACACTCTTTCCCTACACGACGCTCTTCCGATCTCAAAGGATAAACGGGCTGAGA |
| Dmd-OT6 2nd R | GTGACTGGAGTTCAGACGTGTGCTCTTCCGATCTCCATGAGCATGGGAGATCTTT |
| Dmd-OT7 2nd F | ACACTCTTTCCCTACACGACGCTCTTCCGATCTTAGGGAAACAACACCCTTCAC |
| Dmd-OT7 2nd R | GTGACTGGAGTTCAGACGTGTGCTCTTCCGATCTTAATTCTGCCAGTCCATGAGC |
| Dmd-OT8 2nd F | ACACTCTTTCCCTACACGACGCTCTTCCGATCTGGGCACAGCTGAGGAATTA |
| Dmd-OT8 2nd R | GTGACTGGAGTTCAGACGTGTGCTCTTCCGATCTCCAAGCACCACTACCAGATT |
| Dmd-OT9 2nd F | ACACTCTTTCCCTACACGACGCTCTTCCGATCTCACAAAGGATAAATAGGCTGAGAAAG |
| Dmd-OT9 2nd R | GTGACTGGAGTTCAGACGTGTGCTCTTCCGATCTCATGGGAGATCTTTCCGTCTTC |
| Dmd-OT10 2nd F | ACACTCTTTCCCTACACGACGCTCTTCCGATCTAGTAGCTTTCTGCTATGAAAGGAT |
| Dmd-OT10 2nd R | GTGACTGGAGTTCAGACGTGTGCTCTTCCGATCTACAATCCATGAACATGGGAGAT |
| Dmd-OT11 2nd F | ACACTCTTTCCCTACACGACGCTCTTCCGATCTTAAGAAGCGGCCCAGGA |
| Dmd-OT11 2nd R | GTGACTGGAGTTCAGACGTGTGCTCTTCCGATCTGTGGGCAATAAACAAGCCATTTA |
| Dmd-OT12 2nd F | ACACTCTTTCCCTACACGACGCTCTTCCGATCTCGATGATCCTGAGAGTGGAAAT |
| Dmd-OT12 2nd R | GTGACTGGAGTTCAGACGTGTGCTCTTCCGATCTACCCAAATTGTATAACTGAAGCAG |
| Dmd-OT13 2nd F | ACACTCTTTCCCTACACGACGCTCTTCCGATCTCTCAAAGAATAAACAGGCTGAGAAA |
| Dmd-OT13 2nd R | GTGACTGGAGTTCAGACGTGTGCTCTTCCGATCTTAATCCTGCCAAGCCATGAG |
| Dmd-OT14 2nd F | ACACTCTTTCCCTACACGACGCTCTTCCGATCTGAGCTGAATGCCTCTTCCTT |
| Dmd-OT14 2nd R | GTGACTGGAGTTCAGACGTGTGCTCTTCCGATCTTTCACGTCTGGGAGTCTACA |
| Dmd-OT15 2nd F | ACACTCTTTCCCTACACGACGCTCTTCCGATCTAGATCAGGAGCGCACTAGAA |
| Dmd-OT15 2nd R | GTGACTGGAGTTCAGACGTGTGCTCTTCCGATCTATGACCACATCCAGCCTTATTT |
| Dmd-OT16 2nd F | ACACTCTTTCCCTACACGACGCTCTTCCGATCTGAACTTAAAGCACAGAATCAAAGG |
| Dmd-OT16 2nd R | GTGACTGGAGTTCAGACGTGTGCTCTTCCGATCTCATTGCTAAGGACGATGGC |
| Dmd-OT17 2nd F | ACACTCTTTCCCTACACGACGCTCTTCCGATCTGCACCTCCCATAACCGTAAA |
| Dmd-OT17 2nd R | GTGACTGGAGTTCAGACGTGTGCTCTTCCGATCTTAGGGAGGTCAGACATACACTT |
| Dmd-OT18 2nd F | ACACTCTTTCCCTACACGACGCTCTTCCGATCTGGATTCTTGTATTTCTGGCATCTG |
| Dmd-OT18 2nd R | GTGACTGGAGTTCAGACGTGTGCTCTTCCGATCTGCCGGTCTTAGGTCCTATATCT |
| Dmd-OT19 2nd F | ACACTCTTTCCCTACACGACGCTCTTCCGATCTAGAAACCAGGGAAACAACAC |
| Dmd-OT19 2nd R | GTGACTGGAGTTCAGACGTGTGCTCTTCCGATCTCCTACTAATTCATAAGCATGGAAGA |
| Dmd-OT20 2nd F | ACACTCTTTCCCTACACGACGCTCTTCCGATCTGAGCATGATCCATACAAGATATATACAC |
| Dmd-OT20 2nd R | GTGACTGGAGTTCAGACGTGTGCTCTTCCGATCTTCCACTTGCCATTCTCACAG |
| Dmd-OT21 2nd F | ACACTCTTTCCCTACACGACGCTCTTCCGATCTAGGGAAACAGCAGAATGGATAAG |
| Dmd-OT21 2nd R | GTGACTGGAGTTCAGACGTGTGCTCTTCCGATCTTGCTGCTGGACTGTATGTAATG |
| Dmd-OT22 2nd F | ACACTCTTTCCCTACACGACGCTCTTCCGATCTGGATAAAGAGTCTGAGAAAGAAATTGG |
| Dmd-OT22 2nd R | GTGACTGGAGTTCAGACGTGTGCTCTTCCGATCTCCAACCATGAGCATGAGAGT |
| Dmd-OT23 2nd F | ACACTCTTTCCCTACACGACGCTCTTCCGATCTCTCAAAGGATAAACAGGTTGGTAAAG |
| Dmd-OT23 2nd R | GTGACTGGAGTTCAGACGTGTGCTCTTCCGATCTCAAACCATGAGCATGGGAGATA |
| Dmd-OT24 2nd F | ACACTCTTTCCCTACACGACGCTCTTCCGATCTCATCCGTCTGCCTCCTTT |
| Dmd-OT24 2nd R | GTGACTGGAGTTCAGACGTGTGCTCTTCCGATCTCAACCAGGACATCTTCCTACTATC |
| Dmd-OT25 2nd F | ACACTCTTTCCCTACACGACGCTCTTCCGATCTGGGATTAAAGGCATGCACAAC |
| Dmd-OT25 2nd R | GTGACTGGAGTTCAGACGTGTGCTCTTCCGATCTACGGTTATTTGGCAGGAACA |
| Dmd-OT26 2nd F | ACACTCTTTCCCTACACGACGCTCTTCCGATCTCTCCCTGTTGCTGATGAAGATAA |
| Dmd-OT26 2nd R | GTGACTGGAGTTCAGACGTGTGCTCTTCCGATCTAGCCAAAGTTCCTTGGGTAAA |
| Dmd-OT27 2nd F | ACACTCTTTCCCTACACGACGCTCTTCCGATCTTTCCTGAGTATCTCTGGATAGC |
| Dmd-OT27 2nd R | GTGACTGGAGTTCAGACGTGTGCTCTTCCGATCTGTGACAGCAATTCCATTTGTAAG |
| Dmd-OT28 2nd F | ACACTCTTTCCCTACACGACGCTCTTCCGATCTGCTGGGAAAGAAATTAGGGAAAC |
| Dmd-OT28 2nd R | GTGACTGGAGTTCAGACGTGTGCTCTTCCGATCTCAATCCATGAGCATGGGAAATC |
| Dmd-OT29 2nd F | ACACTCTTTCCCTACACGACGCTCTTCCGATCTTTTATAACACATGGTTGGCTCCT |
| Dmd-OT29 2nd R | GTGACTGGAGTTCAGACGTGTGCTCTTCCGATCTTGTGGGTTCTAAAGAAGTGATGG |
| Dmd-cDNA On 2nd F | ACACTCTTTCCCTACACGACGCTCTTCCGATCTAGAGTATCAAACCAACATCATTACC |
| Dmd-cDNA On 2nd R | GTGACTGGAGTTCAGACGTGTGCTCTTCCGATCTTCAATTTGAGGCTGAAGAGC |
